# Inflammasome adaptor ASC promotes sustained neuroinflammation and mild cognitive impairment in a closed-head injury model

**DOI:** 10.1172/JCI199818

**Published:** 2026-02-24

**Authors:** Tao Li, Sergio Castro-Gomez, Pablo Botella Lucena, Ana Vieira-Saecker, Stephanie Schwartz, Yingying Ding, Yushuang Deng, Maling Gou, Valentin Stein, Douglas T. Golenbock, Eicke Latz, Michael T. Heneka

**Affiliations:** 1 Clinic of Parkinson, Sleep and Movement Disorders, Center for Neurology, and; 2 Institute of Physiology II, University Hospital Bonn, University of Bonn, Bonn, Germany.; 3 Luxembourg Centre for Systems Biomedicine (LCSB), University of Luxembourg, Belvaux, Luxembourg.; 4 German Center for Neurodegenerative Diseases (DZNE), Bonn, Germany.; 5 Department of Biotherapy, Cancer Center and State Key Laboratory of Biotherapy, West China Hospital, Sichuan University, Chengdu, PR China.; 6 Division of Infectious Diseases and Immunology, University of Massachusetts Medical School, Worcester, Massachusetts, USA.; 7 Institute of Innate Immunity, University Hospital Bonn, Bonn, Germany.; 8 Centre of Molecular Inflammation Research, Norwegian University of Science and Technology, Trondheim, Norway.; 9 Deutsches Rheuma-Forschungszentrum (DRFZ), Berlin, Germany.

**Keywords:** Inflammation, Neuroscience, Dementia, Innate immunity, Transcriptomics

## Abstract

Mild traumatic brain injury (mTBI) from a closed-head injury (CHI) can lead to prevalent neuropsychiatric disorders, including mood disorders and an increased risk for neurodegenerative diseases and dementia. Inflammasomes are molecular complexes crucial for neuroinflammation and secondary damage after trauma, however their role in mild CHI (mCHI) is poorly understood. In this study, we investigate the cellular expression of inflammasome-related genes and their functional significance in CHI models. Single-cell RNA-seq analysis of cortical tissue after trauma revealed selective expression of *Asc* (also known as *Pycard*), which encodes the inflammasome adaptor apoptosis-associated Speck-like protein containing a caspase recruitment domain (ASC), predominantly in microglial clusters. Sustained upregulation of inflammasome-related proteins, microglia activation, and astrocyte reactivity persisted up to 21 days in a model for mTBI, with significant reduction of this pattern in *Asc^–/–^* mice. Importantly, mild cognitive impairment induced after mCHI was largely abrogated in *Asc^–/–^* mice. These findings suggest that ASC, as the primary inflammasome adaptor, plays a critical role in sustaining neuroinflammation and contributes to cognitive deficits after mCHI. This study provides insights into the molecular neuroinflammatory mechanisms underlying CHI, potentially informing future therapeutic strategies.

## Abstract 2

<span>Our study shows that the inflammasome adaptor ASC drives long-lasting brain inflammation and cognitive problems after mild head injury, highlighting a potential therapeutic target for recovery</span>

## Graphical abstract

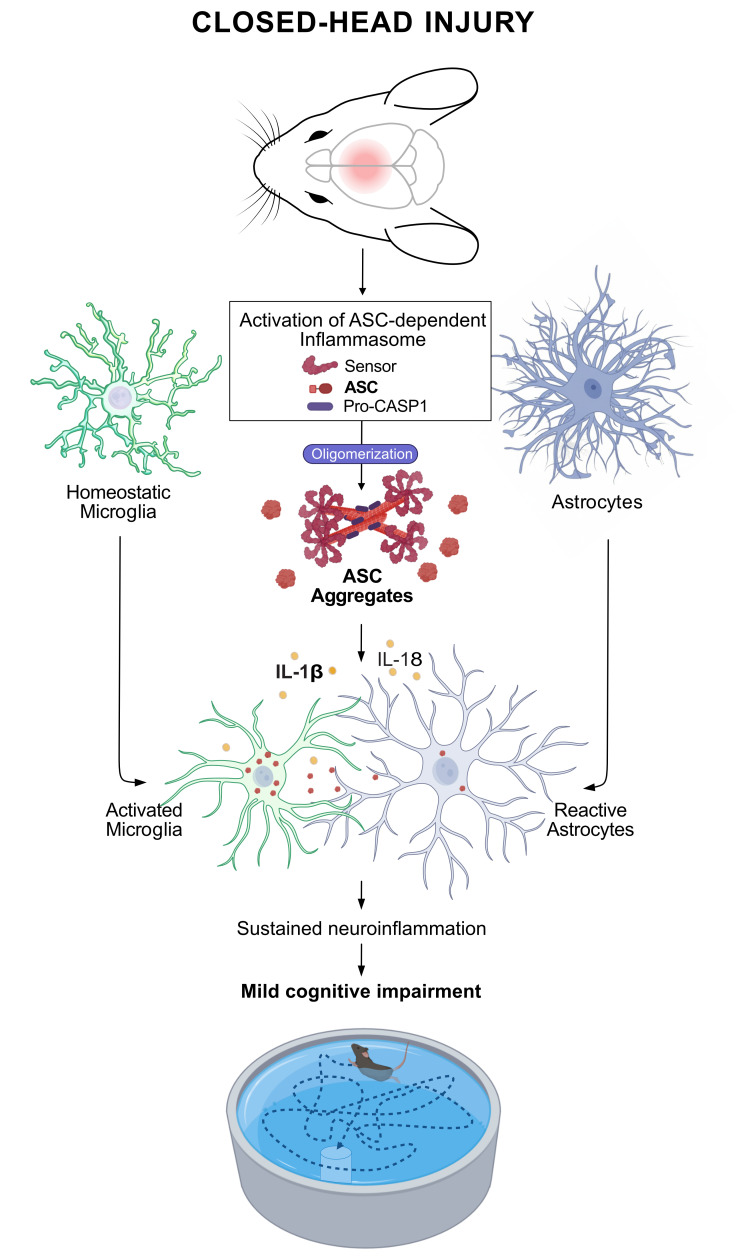

## Introduction

Traumatic brain injury (TBI) represents a significant global health challenge and is considered a silent epidemic, affecting individuals across all demographics and age groups. Every year, TBI affects approximately 1.5 million people in both the European Union (EU) and the United States. In the EU, these injuries are linked to approximately 57,000 deaths and 1.5 million hospital admissions (1), while in the United States, they cause roughly 50,000 deaths, 230,000 hospitalizations with survival, and 80,000–90,000 new cases of long-term disability (2). The majority of cases are clinically classified as mTBI (mTBI), typically resulting from closed-head injuries (CHIs) caused by falls, traffic accidents, violence, contact sports, or military actions (3, 4). Although mTBI symptoms can recover within days or weeks, up to 20% of individuals experience persistent physical, cognitive, and behavioral impairments that lead to a reduced quality of daily life and an elevated risk of neuropsychiatric disorders, including mood disorders and dementia (5–7). Despite its prevalence, the underlying pathophysiology of mTBI resulting from CHI remains poorly understood, posing great challenges for early clinical diagnosis and timely intervention.

Activated microglia and reactive astrocytes play crucial roles in innate immune responses and secondary damage following TBI. Prolonged activation of these cells impairs debris clearance, contributing to neuroinflammation, exacerbating neuronal dysfunction and damage, and promoting abnormal protein aggregation (8–10). Advances in imaging techniques have allowed the in vivo visualization of glial reactivity after CHI, emphasizing the involvement of microglia and astrocytes in the inflammatory cascade that contributes to the development of post-traumatic disorders (11–13). A key element in initiating and maintaining innate immune responses of the brain is inflammasome activation and signaling. Inflammasomes are oligomeric protein scaffolds rapidly activated by damage-associated molecular patterns (DAMPs), and organized as a tripartite complex: a cytosolic danger sensor such as the NLR family pyrin domain–containing protein 3 (NLRP3), a unique adaptor protein called apoptosis-associated speck-like protein containing a caspase recruitment domain (ASC), and a proteolytic effector caspase 1 (CASP1) (14). ASC, an intracellular adaptor protein common to almost all inflammasomes, oligomerizes into highly cross-linked macromolecular assemblies, forming visible ASC aggregates. The fibrillar ASC acts as a molecular platform to recruit pro-CASP1, which optimizes signal transduction via proximity-induced CASP1 activation. Activated CASP1 catalyzes the maturation of pro-forms of IL-1β and IL-18 and mediates the proteolytic activation of gasdermin D (GSDMD). Cleaved GSDMD oligomerizes and forms pores in the plasma membrane, allowing the release of IL-1β, IL-18, and other proinflammatory factors including ASC aggregates. The detection of tissue ASC ensembles is considered a typical hallmark of inflammasome activation (15). Activation of the inflammasome in CHI is likely driven by the well-described 2-signal mechanism initiated by DAMPs released from injured or dying brain cells. The first signal involves priming, in which DAMPs are recognized by pattern recognition receptors (PRRs) such as TLRs. This recognition activates the NF-κB pathway, leading to increased transcription of inflammasome genes, including *Nlrp3* and *Asc*. ​ The second signal, or activation step, is triggered by various cellular stressors and further DAMPs generated during CHI. Key activators include potassium ion (K^+^) efflux (16), mitochondrial dysfunction with increased ROS production (17), extracellular ATP signaling via P2X7 receptors (18), and lysosomal disruption often resulting from phagocytosis of neuronal and myelin debris (19). Additional contributors include alterations in intracellular calcium levels, osmotic imbalances (20), and stress of organelles such as lysosomes (21) and endoplasmic reticulum (22), all converging to promote assembly of the inflammasome complex.

In humans, some inflammasome proteins are detected in the blood and cerebrospinal fluid after TBI, and circulating levels of some proteins such as ASC and CASP1 have been proposed as biomarkers for determining injury severity (23–25). Furthermore, inflammasome activators, including NLRP1 (26), NLRP3 (27, 28), and absent in melanoma 2 (AIM2) (29), presumably contribute to the innate immune response and functional disability in mouse models of TBI. These studies primarily focused on inflammasome activation in models of moderate-to-severe TBI, such as the commonly used controlled cortical impact (CCI), in which ASC has been hypothesized to be dispensable (30). At the same time, the contribution of these inflammasome activators to mild CHI (mCHI) remains largely unexplored. In the present study, we hypothesized that ASC ensembles contribute actively to the secondary damage following CHI. We aimed to investigate the role of inflammasomes in neuroinflammatory responses and subsequent cognitive functions by analyzing a well-established single-cell RNA-seq (scRNA-seq) dataset of cortical cells from mice subjected to CHI (31) and by using an additional CHI mouse model that induces mild cognitive impairment (32). Our scRNA-seq data analysis revealed subacute expression of *Asc* (also known as *Pycard*) in cortical cells from mice subjected to midline fluid percussion injury (mFPI), particularly in microglia subpopulations. In concordance with this analysis, following CHI, we observed sustained upregulation of inflammasome-related proteins, including NLRP3, ASC, CASP1, and IL-1β that persisted up to 21 days post injury (dpi). This expression pattern was markedly reduced in *Asc^–/–^* mice. Moreover, *Asc^–/–^* mice were protected from the mild cognitive impairment induced by our CHI model. We also observed ASC-dependent microglial and astrocytic reactivity and their cellular interactions that persisted over time, accompanied by the upregulation, aggregation, and redistribution of ASC protein. Our findings identify ASC, the primary inflammasome adaptor, as a key molecule in sustaining neuroinflammation and contributing to cognitive deficits after CHI.

## Results

### Subacute Asc expression in microglia subpopulations after CHI.

**Figure 1:**
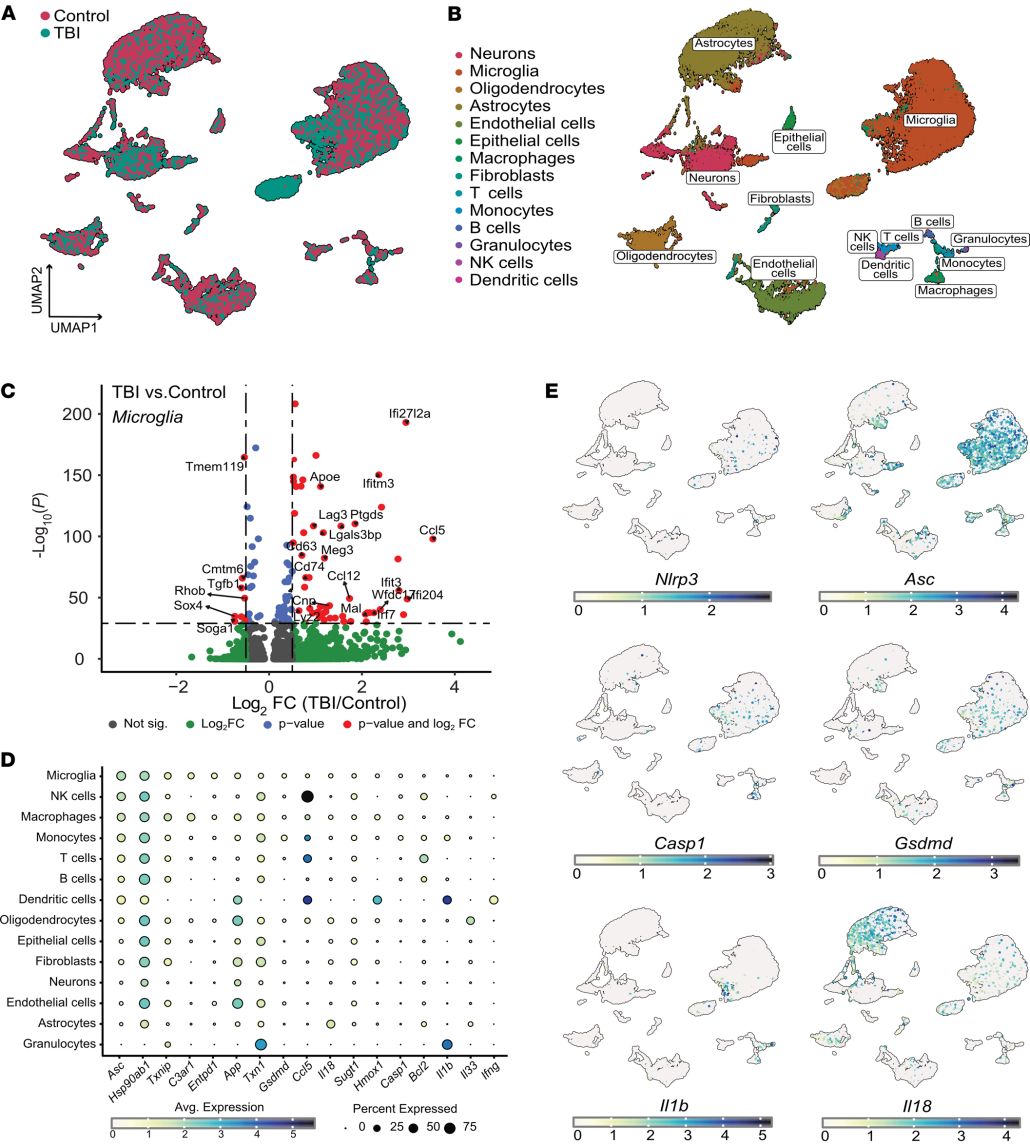
Subacute *Asc* expression in microglia in CHI model. (**A** and **B**) scRNA-seq analysis of murine brain cell suspensions 7 days after mFPI. (**A**) UMAP visualization shows clustering of different cell populations, with mFPI and control samples overlaid. (**B**) Annotated cell types, including microglia, macrophages, and other immune and neural cells after cluster identification. (**C**) Volcano plot depicting log_2_ fold change (TBI/control) versus –log_10_ *P*-value for microglial genes in mFPI compared to controls. Genes are color-coded: gray (not significant), green (significant by log fold change), blue (significant by *P*-value), red (significant by both *P*-value and log fold change). Dashed lines indicate significance thresholds. Not sig., not significant. (**D**) Dot plot expression analysis showing the percentage of cells expressing key inflammasome-related genes across various brain cell types, highlighting *Asc* expression in microglia, NK cells, macrophages, and monocytes. Avg., average. (**E**) UMAP projection of colored cells relative to the expression of the canonical inflammasome-related genes *Nlrp3*, *Pycard* (*Asc*), *Casp1*, *Il1b*, *Il18*, and *Gsdmd*.

To examine the cellular expression of genes coding for inflammasome-related proteins after CHI, our first approach was to conduct single-cell transcriptomics analysis. We chose to reexamine a publicly available scRNA-seq dataset from dissociated cortical cells 7 days (7 dpi) after mFPI (31). mFPI mimics several relevant features of CHI, including diffused trauma without localized lesions or hemorrhages, and closely models mild-to-moderate TBI (33). After quality control, we analyzed 14,688 control cells and 10,745 cells in the mFPI group (Figure 1A and Supplemental Figure 1A; supplemental material available online with this article; https://doi.org/10.1172/JCI199818DS1). The complex multidimensional dataset was transformed and visualized using the nonlinear dimensionality reduction technique uniform manifold approximation and projection (UMAP) (Figure 1, A, B, and E). Examination of cellular transcriptional identities at the single-cell level uncovered 22 distinct cellular clusters. Cellular subpopulation labels were assigned via automated cell-type annotation per metadata sample using SingleR (34) (Supplemental Figure 1B). Cells identified as microglia were especially enriched in this dataset (Figure 1B). Differential gene expression analysis of this subset of cells showed a typical disease-associated microglial signature after TBI, with overexpression of type 1 IFN genes (such as *Ifi27l2a*, *Ifitm3*, *Ifit3*, *Irf7*) and of *Apoe*, as well as reduced expression of homeostatic genes such as *tmem119* or *tgfb1* (Figure 1C).

We then evaluated, for each predicted cellular subpopulation, the expression of genes annotated to pathways related to inflammasome activation (pathcards.genecards.org [ref. 35], Supplemental Figure 1C). Notably, we found that *Asc* was one of the genes expressed more widely in clusters represented by microglia, macrophages, and other peripheral infiltrating immune cells (Figure 1, D and E, and Supplemental Figure 1, C and D). These clusters showed a reduced percentage of cells expressing genes such as *Gsdmd, Casp1, Il1b*, or *Il18*, which code for inflammasome effectors (Figure 1, D and E). Surprisingly, we found a very low percentage of cells expressing genes such as *Nlrc4*, *Nlrc5*, *Nlrp1b*, *Nlrp3*, or *Aim2*, which code for inflammasome activators Supplemental Figure 1C). These results suggest that the subacute and prolonged expression of the adaptor protein ASC, especially in microglia, may play a central role in the sustained innate immune response and pathophysiology of CHI.

### ASC contributes to the sustained expression and processing of inflammasome mediators following mCHI.

**Figure 2:**
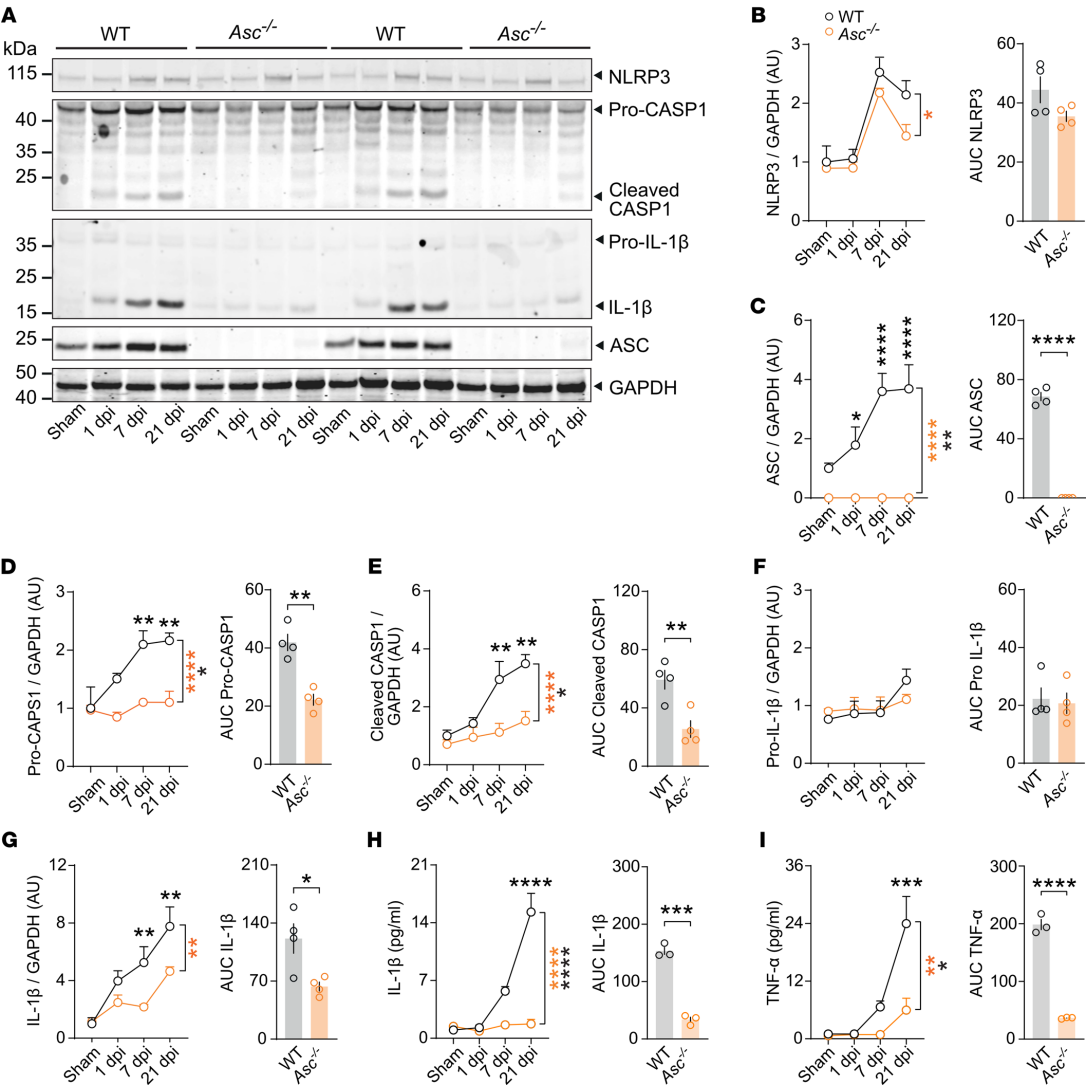
ASC contributes to the upregulation of inflammasome mediators following CHI. (**A**) Immunoblot detection and (**B**–**G**) densitometric semiquantification of the inflammasome-related proteins NLRP3, ASC, CASP1, and IL-1β at 1, 7, and 21 dpi in contusional cortices from WT and *Asc^-–/–^* mice following sham surgery or CHI (left panels *n* = 4 per group and time point). (**H** and **I**) IL-1β and TNF-α levels in brain lysates from WT and *Asc^–/–^* mice at 1, 7, and 21 dpi. *n* = 3–4 mice per group and time point. Right panels in **B**–**I** show integrated relative concentrations (AUC). *P < 0.05, **P < 0.01, ***P < 0.001, and ****P < 0.0001, by ordinary 2-way ANOVA with Bonferroni’s test (**B**–**I**, left panels) and Student’s *t* test (**B**–**I**, right panels). Data are presented as the mean ± SEM.

To unveil the pathophysiological role of inflammasomes and specifically of ASC as the main inflammasome adaptor and abundantly expressed gene in microglia after CHI, we adopted an electromagnetic CHI procedure as the main model for mTBI (32). In this model, a controlled cortical impact injury is delivered directly to the midline surface of the skull using a 5 mm diameter tip at a specified depth and velocity (Supplemental Figure 2A). We found mild motor, reflex, or reaction deficits at 1 and 7 dpi when the mice were evaluated using the Revised Neurobehavioral Severity Scale (NSS-R) for rodents (36) (Supplemental Figure 2B). To examine the expression of inflammasome-related proteins after CHI, we performed immunoblot analysis of cortices from WT and *Asc^–/–^* mice that underwent either sham or CHI surgery. We analyzed and semiquantified the protein levels of NLRP3, ASC, CASP1, and IL-1β at 1, 7, and 21 dpi (Figure 2, A–I). Our results revealed sustained upregulation of these proteins up to 21 days after CHI in WT mice. Notably, mice with a constitutive genetic deficiency of the *Asc* gene (*Asc^–/–^*) showed a significant deficit in the expression and cleavage of CASP1 (Figure 2, D and E) and of IL-1β over time (Figure 2G) following CHI. ELISA measurements further demonstrated significantly lower soluble levels of IL-1β and TNF-α in *Asc^–/–^* mice (Figure 2, H and I), particularly at later time points. We additionally evaluated the levels of cleavage of caspase 8 (CASP8), a proposed noncanonical enzyme involved in IL-1β processing (37) (Supplemental Figure 2, C and D), and its downstream effector caspase 3 (CASP3), as indirect measurement of apoptotic pathways (38) (Supplemental Figure 2, C and E). Consistent with the reduction in soluble IL-1β, *Asc^–/–^* mouse cortices showed significantly reduced levels of cleaved CASP8 up to 21 days after CHI (Supplemental Figure 2D), which suggests parallel activation of canonical and noncanonical pathways of IL-1β processing, both of which appeared largely dependent on ASC. Additionally, the cleavage of CASP3 was significantly reduced in *Asc^–/–^* mice (Supplemental Figure 2E), suggesting a strong neuroprotective effect and complex crosstalk involving the activation of multiple types of cell death influenced by inflammasome signaling (39). Further colocalization analysis of cleavage CASP3 immunostaining with the ionized calcium-binding adaptor molecule 1 (Iba1^+^) and glial fibrillary acidic protein (GFAP^+^) on cortices from WT mice revealed that cleaved CASP3 was predominantly localized in both the cytoplasm and nucleus of Iba1^+^ cells, likely microglia, whereas its expression of GFAP^+^ cells, likely astrocytes, was comparatively weaker. These results suggest that CASP3 activation occurred in microglia or infiltrating macrophages, where it may have participated in both apoptotic (nuclear) and nonapoptotic (cytoplasmic) signaling processes, consistent with its reported roles in regulating neuroinflammatory responses (40). Notably, microglia and astrocytes exhibiting stronger nuclear localization of cleaved CASP3 displayed neuroinflammatory morphological features, including nuclear condensation and cell-body shrinkage (Supplemental Figure 2, F and G).

To identify the cellular sources of IL-1β following mCHI, we additionally conducted immunohistochemical staining for IL-1β, Iba1, and GFAP on cortices of WT mice in the 7 and 21 dpi groups (Supplemental Figure 3A). At day 7 after mTBI, we found that IL-1β signal was colocalized with or in the vicinity of GFAP^+^ cells (presumably reactive astrocytes) and with Iba1^+^ cells (likely microglia) (Supplemental Figure 3A). Interestingly, at 21 dpi, the majority of IL-1β was detected in Iba1^+^ cells (microglia or infiltrating macrophages), suggesting a very active role of microglia in the TBI-dependent upregulation and secretion of this cytokine (Supplemental Figure 3, B and C). Our findings thus emphasize the crucial role of inflammasome signaling, with ASC as the common adaptor protein, in sustaining the expression and processing of neuroinflammatory mediator after CHI. Moreover, we identified Iba1^+^ cells, likely microglia, or infiltrating macrophages as the primary cellular sources of IL-1β, especially at later post-injury time points.

### ASC shapes the morphology of Iba1^+^ cells following CHI.

**Figure 3:**
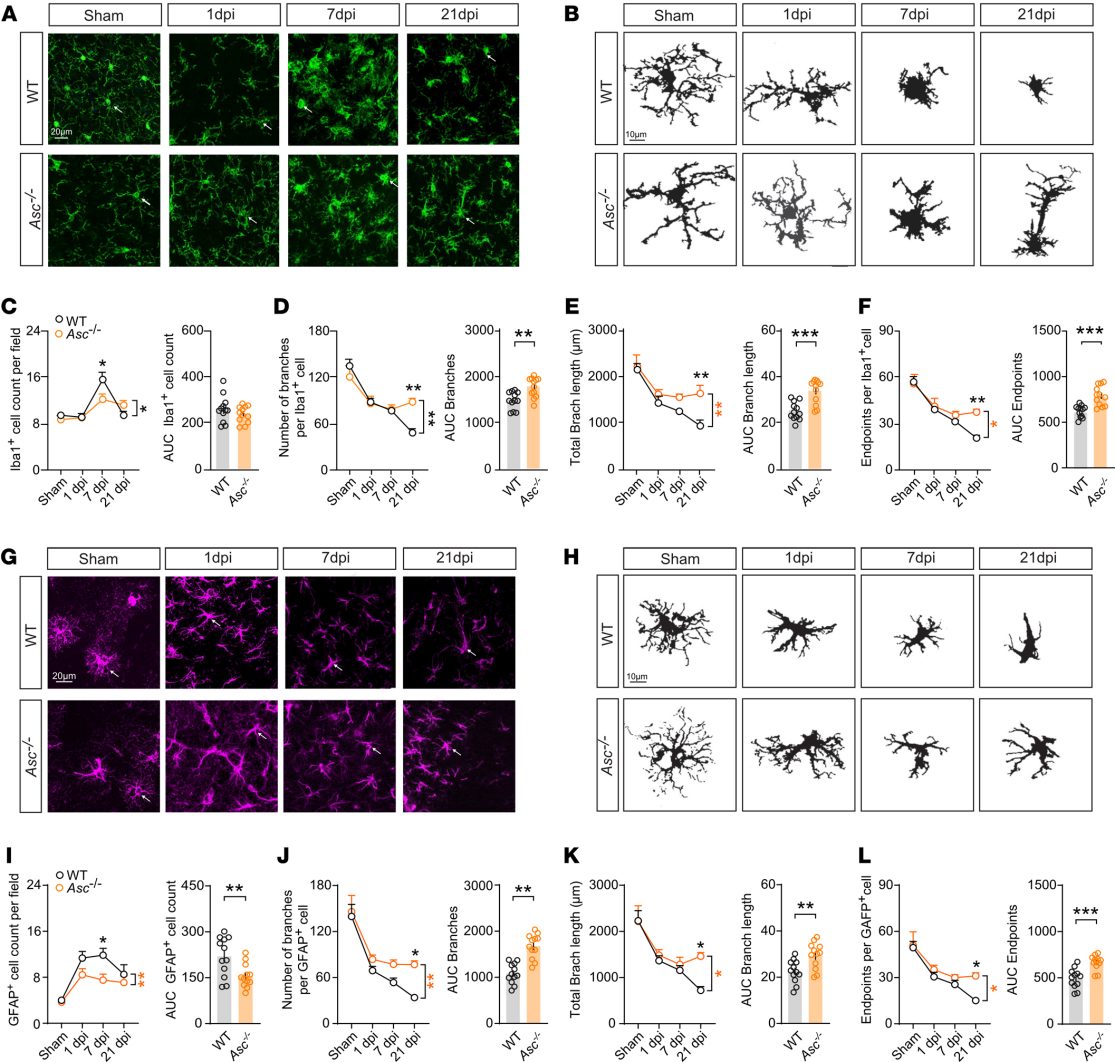
Morphology of Iba1^+^ and GFAP^+^ cells is modulated by ASC following CHI. (**A**) IHC images of Iba1 (green, presumably microglia) staining in cortices from WT and *Asc^–/–^* mice subjected to sham surgery or at 1, 7, and 21 dpi. Arrows indicate representative morphological changes. Scale bar: 20 μm. (**B**) Representative skeletonized Iba1^+^ cells following CHI. Scale bars: 10 μm. Quantitative analysis of (**C**) Iba1^+^ cell counts, (**D**) branch numbers per cell, (**E**) total branch length per cell, and (**F**) endpoint numbers per cell at 1, 7, and 21 dpi for WT and *Asc^–/–^* mice subjected to sham or CHI. (**G**) IHC images of GFAP (magenta, presumably astrocytes) in cortices from WT and *Asc^–/–^* mice at 1, 7, and 21 dpi. Arrows indicate representative morphological changes. Scale bar: 20 μm. (**H**) Representative skeletonized GFAP^+^ cells following CHI. Scale bars: 10 μm. Quantitative analysis of (**I**) GFAP^+^ cell counts (**J**) branch numbers per cell, (**K**) total branch length per cell, and (**L**) endpoint numbers per cell at 1, 7, and 21 dpi for WT and *Asc^–/–^* mice subjected to sham surgery or CHI. *n* = 12 slices from 4 mice per group per time point. **P < 0.01 and ***P < 0.001, by ordinary 2-way ANOVA with Bonferroni’s post hoc test and 2-tailed Student’s *t* test. 27. *P < 0.05, **P < 0.01, ***P < 0.001, and ****P < 0.0001, by ordinary 2-way ANOVA with Bonferroni’s test (**C**–**F** and **I**–**L**, left panels) and Student’s *t* test (**C**–**F** and **I**–**L**, right panels). Data are presented as the mean ± SEM. Right panels show the integrative AUC of each marker over time (dpi).

Microglia exhibit distinct adaptive morphologies in response to mechanical stimuli, and their morphology is considered a proxy for their function (41). To investigate microglia architecture after CHI, we performed immunofluorescence staining for Iba1 and skeleton analysis (42) to examine cell morphology in cortices of WT and *Asc^–/–^* mice at 1, 7, and 21 dpi (Figure 3, A and B). First, we observed that *Asc^–/–^* mice consistently exhibited lower Iba^+^ cell counts compared with WT mice at 7 dpi (Figure 3C), suggesting decreased microgliosis in the subacute CHI phase. Next, we quantified the normalized changes in the morphology of Iba1^+^ cells based on cell count. The morphological alterations, including the number of branches per cell, endpoints per cell, and branch length per cell, substantially decreased after injury in WT mice, particularly at 21 dpi. In contrast, *Asc^–/–^* mice showed a significant preservation of these morphological features at 21 dpi compared with WT mice (Figure 3, D–F). In conclusion, our observations suggest a wave of microgliosis in the subacute phase and revealed a progressive debranching of Iba1^+^ cells over time following injury. Notably, the lack of ASC demonstrated protection against these sustained cellular morphological alterations, particularly at later time points.

### ASC regulates the reactivity of GFAP^+^ cells following CHI.

Previous studies have provided evidence that morphological changes in astrocytes are intricately linked to microglia activation during inflammation-induced responses (43). To examine reactive astrocytes and their morphological alterations following mCHI, we performed morphological analysis of GFAP^+^ cells in contusional layers 1 and 2/3 of the retrosplenial and primary motor cortices, using the same method as for Iba1^+^ cells (Figure 3, G and H). We initially observed a noteworthy increase in the GFAP^+^ cell count at 1, 7, and 21 dpi in WT mice (Figure 3I), confirming increased reactive astrogliosis in our model. *Asc* deficiency exerted a genotype-related effect in the cell count similar to what we observed in Iba1^+^ cells, showing significantly lower numbers, especially at 7 dpi (Figure 3I). Subsequently, we quantified normalized morphological changes in GFAP^+^ cells on the basis of cell counts. The branches per cell, endpoints per cell, and length of branches per cell were significantly decreased after CHI, particularly at 21 dpi (Figure 3, J–L). In contrast, *Asc^–/–^* mice showed preserved morphological features at 21 dpi compared with WT mice (Figure 3, J–L). Interestingly, at 21 dpi, we observed clusters of aggregated GFAP^+^ cells, indicating post-injury spatial reorganization. Similar morphological changes were evident in both microglia and reactive astrocytes after injury, suggesting a potential correlation or interactions between these 2 glial cell populations.

### Iba1^+^ and GFAP^+^ cell interactions are modulated by ASC following CHI.

**Figure 4:**
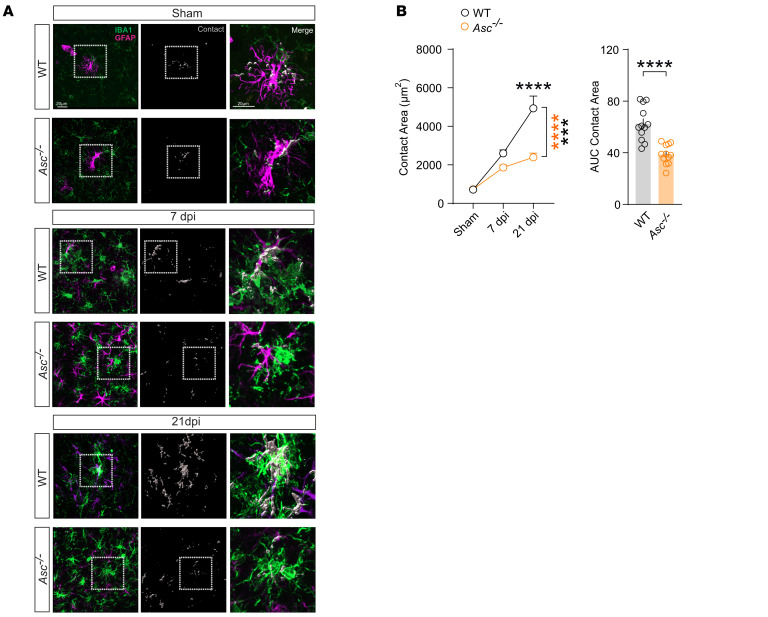
Cellular interactions of microglia and reactive astrocytes are modulated by ASC following CHI. (**A**) IHC images for Iba1 (green) and GFAP (magenta) staining in cortices of WT and *Asc^–/–^* mice following sham surgery and 7 and 21 dpi. Surface-surface colocalization analysis after 3D reconstruction was used to estimate contact areas between Iba1^+^ cells and GFAP^+^ cells. Rectangles highlight examples of glial surface-surface colocalization, shown in high-magnification views. Scale bars: 20 μm. (**B**) Quantification of contact areas between Iba1^+^ cells and GFAP^+^ cells at 7 and 21 dpi in WT and *Asc^–/–^* mice subjected to sham surgery or CHI. *n* = 12 slices from 4 mice per group per time point. ***P < 0.001 and ****P < 0.0001, by ordinary 2-way ANOVA with Bonferroni’s post hoc test (left panel) and 2-tailed Student’s *t* test (right panel). Right panel shows the integrative AUC of each marker over time (dpi).

To investigate the interactions between reactive astrocytes and microglia in the contusional cortex following injury, we performed surface-surface colocalization analysis of the contact areas after 3D reconstruction of confocal images from GFAP- and Iba1-immunostained tissues (Figure 4A). We observed a significant increase in contact area between reactive astrocytes and microglia at 7 dpi, reaching a peak at 21 dpi in WT mice (Figure 4B). Notably, the contact area was significantly reduced at 7 and 21 dpi in *Asc^–/–^* mice (Figure 4B), which may be explained by the deficient microglia activation and reactive astrogliosis observed in our previous analysis. These findings, together with our expression analysis, suggest that inflammasome ensembles and neuroinflammatory processing are essential for sustained microglial and astrocytic responses and cellular interactions after mCHI.

### Genetic deficiency in the Asc gene provides protection against mild cognitive impairment following mCHI.

**Figure 5:**
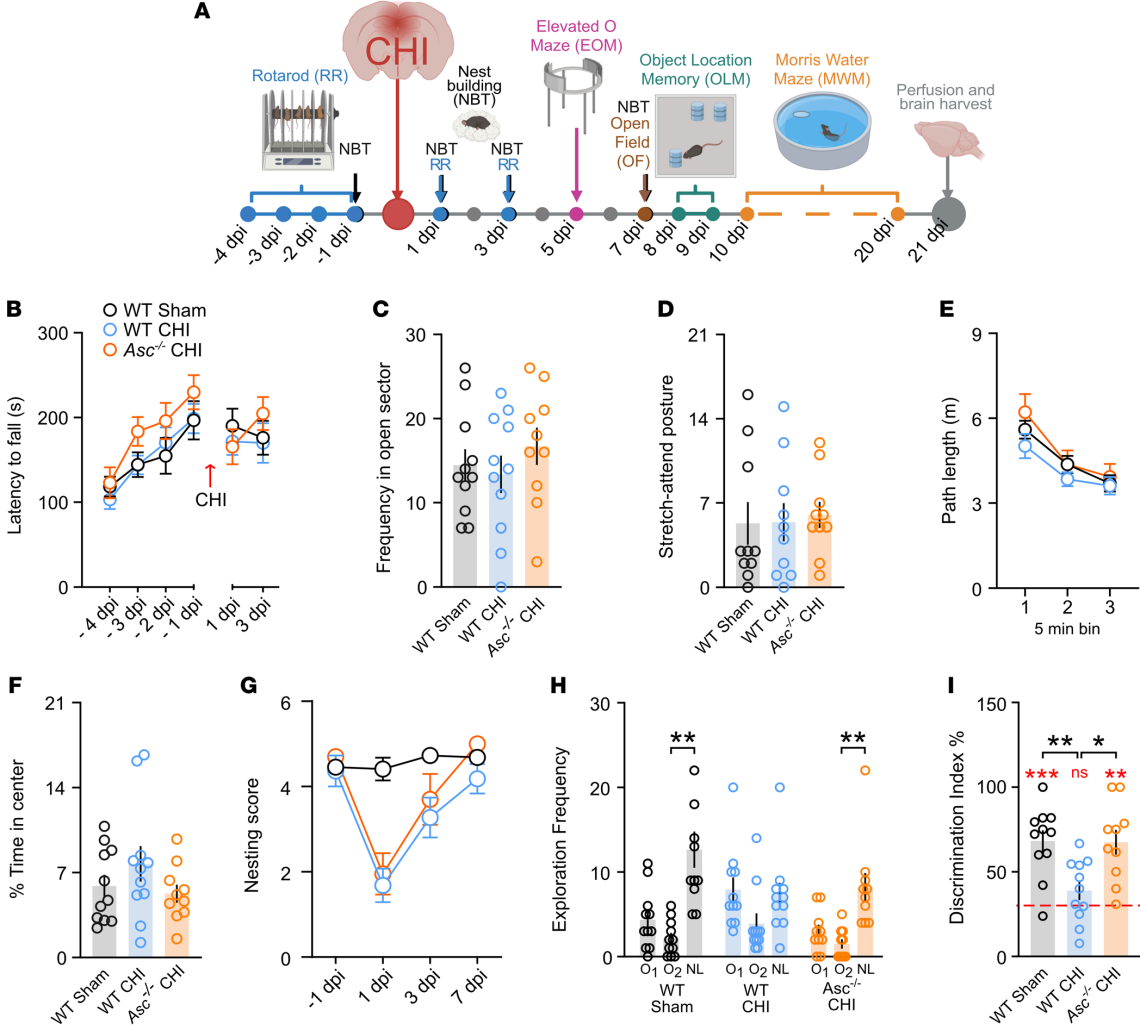
*Asc^–/–^* mice show preserved short-term spatial memory following mCHI. (**A**) Experimental timeline of the behavioral test battery for cognitive assessment following CHI. Rotarod (RR) performance was evaluated between –4 and –1 days before injury and at 1 and 3 dpi. NBTs were conducted at day –1 day before injury and 1, 3, and 7 dpi. The EOM was conducted at 5 dpi, the OF test at 7 dpi, the OLM test at 8 and 9 dpi, and the MWM test from 10–20 dpi. (**B**–**G**) Comparable behavioral performances were observed among sham-treated WT mice, WT mice subjected to CHI, and *Asc^−/−^* mice subjected to CHI for (**B**) latency to fall during the rotarod tests, (**C**) frequency in open sectors, (**D**) stretch-attend postures in the EOM, (**E**) path length, and (**F**) percentage of time spent in the center of the OF (*n* = 10–11 mice per genotype; 1-way ANOVA). (**G**) NBT scores (*n* = 10–11 mice per genotype; repeated-measures 2-way ANOVA). (**H** and **I**) *Asc^−/−^* mice subjected to CHI, similar to sham-treated WT mice (**H**) explored 1 of the displaced objects significantly more frequently than the other 2 objects (**P < 0.01, by Friedman test with Dunn’s multiple-comparison test) and (**I**) exhibited a higher discrimination index compared with both the chance level (**P < 0.01 and ***P < 0.001, by 1-sample *t* test vs. 33.3% chance level shown by a red dashed line) and CHI WT mice (*P < 0.05 and **P < 0.01, by 1-way ANOVA with Tukey’s post hoc test).

**Figure 6:**
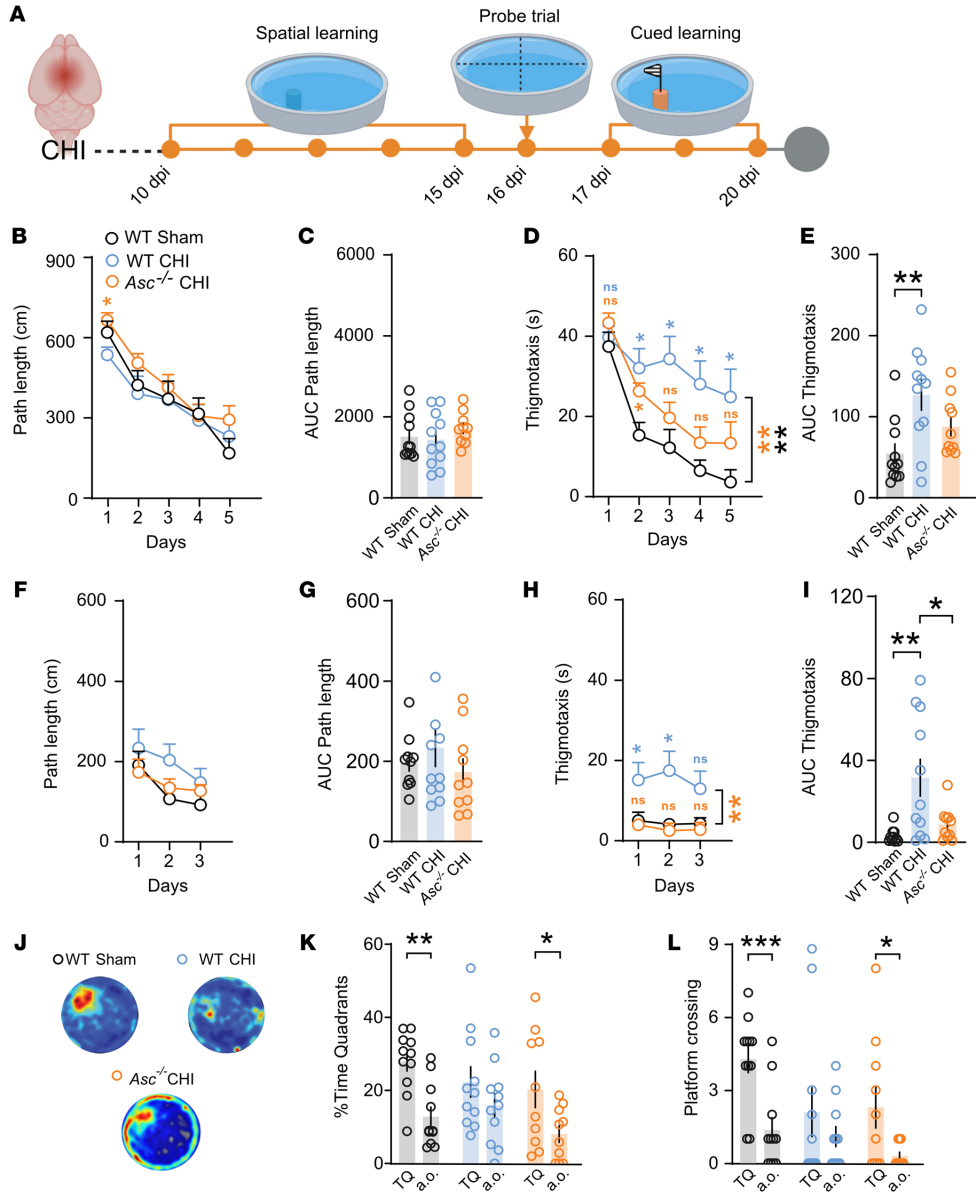
Genetic deficiency of the *Asc* gene protects against mild cognitive deficits in the MWM following mCHI. (**A**) Experimental timeline for cognitive assessment following CHI, including the spatial learning phase (10–15 dpi), the probe trial (16 dpi), and cued learning (17–20 dpi) in the MWM test. (**B**–**E**) MWM performance of WT and *Asc*^–/–^ mice, showing path length and thigmotaxis across spatial (**B**–**E**) and cued (**F**–**J**) training days (*n* = 10–11 mice per genotype. Integrative AUC for path length and thigmotaxis from spatial learning (**C** and **E**) and cued learning phase (**G** and **I**). (**J**) Representative heatmaps mean swimming trajectories and (**K**) quantification of the percentage of time spent in the target quadrant compared with another quadrant (a.o.) or (**L**) platform crossing times in the virtual target platform compared with another virtual platform (a.o.). *n* = 10–11 mice per genotype. *P < 0.05, **P < 0.01, and ***P < 0.001, by repeated-measures 2-way ANOVA with Tukey’s post hoc test (**B**–**I**) and ordinary 1- or 2-way ANOVA with Tukey’s post hoc test (**K** and **L**). Data are presented as the mean ± SEM.

Having demonstrated that *Asc* was predominantly expressed by microglia subpopulations in the subacute phase of CHI, contributing to the sustained inflammasome activation, as well as microglial and astrocytic reactivity following CHI, we next sought to investigate whether genetic removal of the main inflammasome adaptor protein ASC influences cognitive outcomes after mCHI. In humans, a significant number of individuals show transient or persistent cognitive impairment following mTBI, including deficits in memory recall, procedural tasks, executive functions, attention, and emotional symptoms such as heightened anxiety (44). To investigate the role of ASC in neurobehavioral phenotypes following mCHI, we conducted a comprehensive battery of behavioral tests evaluating motor, emotional, and cognitive functions (Figure 5A). Motor coordination and balance were assessed using the rotarod task from 4 days before until 3 dpi. Anxiety-related and exploratory behaviors were measured by the elevated O-maze (EOM) and open-field (OF) tests at 5 and 7 dpi, respectively. Nest-building behavior test (NBT), a sensitive indicator of motivation and stress, was evaluated 1 day before and at 1, 3, and 7 dpi. Short-term spatial memory was examined with the novel object location memory (OLM) test at 7 and 8 dpi. Furthermore, mice were subjected to the Morris Water Maze (MWM) test, a more cognitively demanding paradigm, from 10 to 20 dpi, including a spatial learning phase (10–15 dpi), a 24-hour long-term memory test (16 dpi), and a cued learning phase (17–20 dpi) to assess procedural learning and sensorimotor function (Figure 5A and Figure 6A).

Our mCHI protocol did not induce impairments in latency to fall (Figure 5B) or running speed (Supplemental Figure 4A) in the rotarod test, or in exploratory frequency (Figure 5C and Supplemental Figure 4B) or stretch-attend postures in the open sectors (Figure 5D) of the EOM, or in total path length in the OF (Figure 5E). Injured WT and *Asc^–/–^* mice showed no significant increase in the percentage of time spent in the center (Figure 5F) or in locomotor velocity in the OF (Supplemental Figure 4C). Although mCHI induced transient deficits in nest building at 1 and 3 dpi, no differences were observed between WT and *Asc^–/–^* mice at any assessed time point (Figure 5G).

At 8 and 9 dpi, the experimental groups underwent the OLM test to assess short-term spatial memory. Mice were trained for 2 consecutive days with 3 identical objects placed in a box containing visual cues on the walls. We observed no differences in the exploration frequency of 3 identical objects during the training phase across the experimental groups (Supplemental Figure 4D). One hour after the last training session, 1 object was relocated to a novel position. Mice naturally explored the displaced object for a considerably longer time. mCHI resulted in significant impairment of both the exploration frequency (Figure 5H) and the preference index in the OLM test for WT mice (Figure 5I). These deficits were absent in *Asc^–/–^* mice subjected to mCHI (Figure 5, H and I), indicating a preserved short-term spatial memory.

During the MWM test (Figure 6A), WT mice subjected to mCHI exhibited a daily reduction in path length toward the hidden platform comparable to that seen with WT sham animals (Figure 6, B and C). However, CHI significantly increased thigmotaxis, as reflected by prolonged swimming near the pool walls during the learning phase for injured WT mice (Figure 6, D and E). In the cued version of the task, WT injured mice similarly demonstrated a daily reduction in path length (Figure 6, F and G) and increased thigmotactic behavior (Figure 6, H and I). This procedural deficit was significantly reduced in *Asc^–/–^* mice (Figure 6, D, E, H, and I). Moreover, *Asc^–/–^* mice displayed preserved long-term spatial memory 24 hours after the learning phase, as visually suggested by the density plots (heatmaps) of the swim paths (Figure 6J). This observation was further supported by a significantly greater percentage of time spent exploring the target quadrant (Figure 6K) and by more frequent crossings over the virtual target platform compared with control quadrants or platforms during the probe trial at 16 dpi (Figure 6L). Furthermore, *Asc^–/–^* mice showed slightly higher velocity on the last day of cued learning (Supplemental Figure 4, E and F). Collectively, these data show that CHI induced mild cognitive impairments, mainly as procedural and memory deficits in the OLM and the MWM tests, and these impairments appeared to be largely dependent on ASC expression.

### NLRP3 regulates ASC aggregation and distribution following CHI.

**Figure 7:**
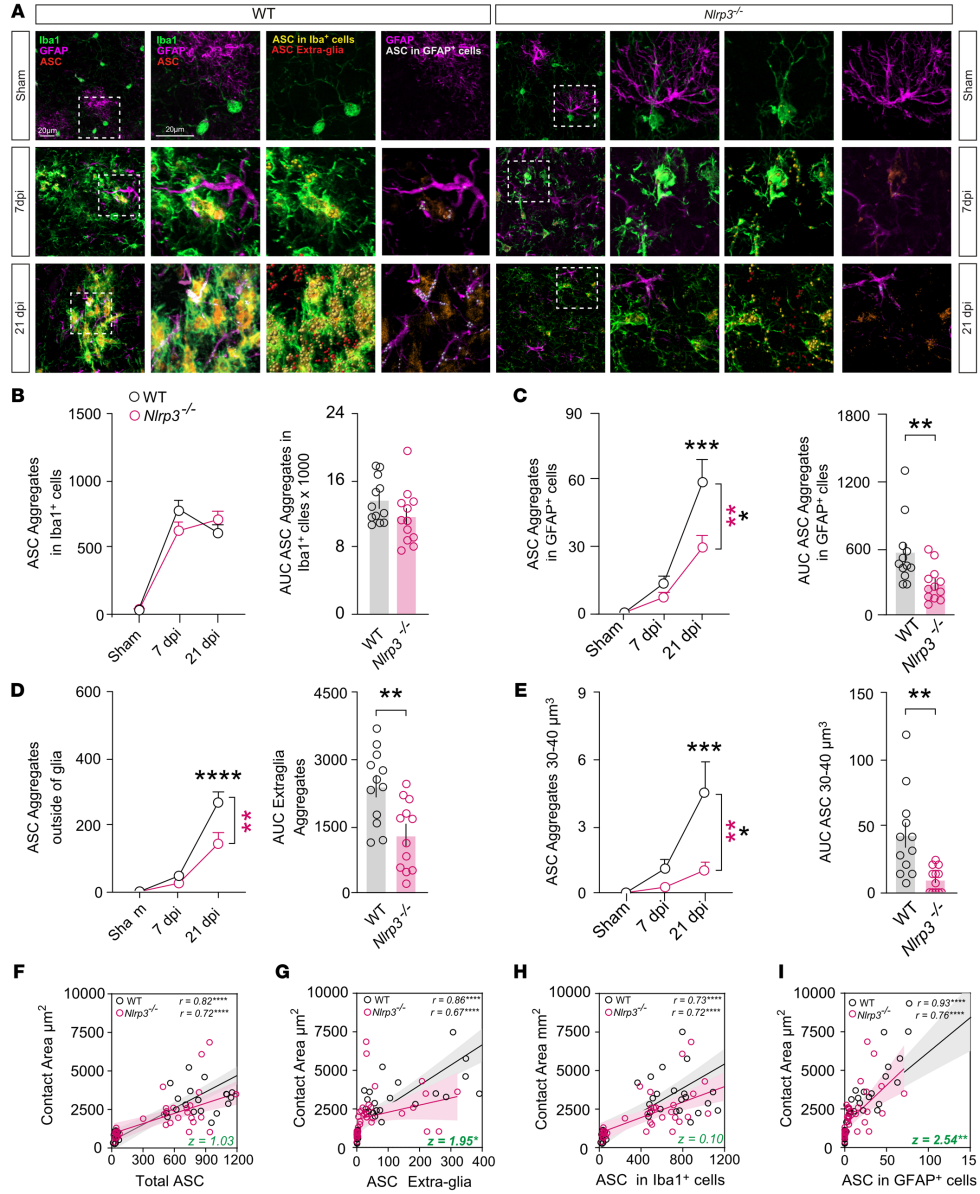
NLRP3 regulates ASC aggregation and distribution following CHI. (**A**) IHC images for Iba1 (green), GFAP (magenta), and ASC (red) staining in cortices of WT and *Nlrp3^–/–^* mice at 7 and 21 dpi. ASC localization in Iba1^+^ cells (yellow spots), GFAP^+^ cells (blue spots), and outside glial cells (red spots) was analyzed using IMARIS. Rectangles highlight examples of ASC distribution in high-magnification views. Scale bars: 20 μm. (**B**–**E**) Quantification of total ASC aggregates (**B**), intracellular ASC aggregates in Iba1^+^ cells (**C**), ASC aggregates outside glial cells (**D**), and ASC aggregates with a volume range of 30–40 μm^3^ (**E**) at 7 and 21 dpi in WT and *Nlrp3^–/–^* mice subjected to sham surgery or CHI. *n* = 12 slices from 4 mice per group per time point. *P < 0.05, **P < 0.01, ***P < 0.001, and ****P < 0.0001, by ordinary 2-way ANOVA with Bonferroni’s post hoc test (**B**–**E**, left panels) and 2-tailed Student’s *t* test (**B**–**E**, right panels). Right panels show integrative AUC of each marker over time (dpi). Data are presented as the mean ± SEM. (**F**–**I**) Spearman’s correlation analysis (*r*) with a linear regression model (black lines represent WT mice; red lines represent *Nlrp3^–/–^* mice) with an interaction term for ASC aggregates (total, within glial cells and outside glial cells) and glial cell contact areas. *n* = 36 slices from 12 mice per genotype. ****P < 0.0001. Differences between WT and *Nlrp3^–/–^* Spearman correlation coefficients (*r*) were calculated using standard Fisher’s *z*-transformation and subsequent comparison (87). *P < 0.05 and **P < 0.01.

The NLRP3 is widely recognized as one of the most relevant inflammasome activators in the context of TBI, with growing evidence indicating its central role in mediating neuroinflammation and secondary brain damage (45, 46). To explore the involvement of NLRP3 in the aggregation of ASC as the main inflammasome adaptor in following CHI, contusional cortical tissue was immunohistochemically stained for Iba1, GFAP, and ASC at 7 and 21 dpi in WT and *Nlrp3^–/–^* mice (Figure 7A). We used a KO-validated monoclonal antibody for ASC detection (Supplemental Figure 5, A and B). Subsequently, we conducted 3D surface reconstruction of Iba1, GFAP, and ASC immunosignal analysis (Figure 7A and Supplemental Figure 5C). We detected a marked increase in the total number of fluorescent ASC aggregates, especially in Iba1^+^ cells (likely microglia) at 7 dpi, similar to the mRNA expression of *Asc* observed in the scRNA-seq data analysis following mFPI (Figure 1B, Figure 7B, and Supplemental Figure 5D). The number of ASC aggregates within Iba1^+^ cells followed the total ASC aggregates pattern (Figure 7B and Supplemental Figure 5, C and D). Notably, *Nlrp3^–/–^* injured mice showed comparable ASC aggregate counts, both overall and within Iba1^+^ cells, compared with WT mice (Figure 7B), but these mice had significantly fewer aggregates in GFAP^+^ cells (presumably reactive astrocytes, Figure 7C). Interestingly, at 21 dpi, the number of ASC aggregates outside Iba1^+^ and GFAP^+^ cells (extra-glia ASC) significantly increased in both WT and *Nlrp3^–/–^* mice (Figure 7D). However, this ensemble count was significantly lower in *Nlrp3^–/–^* mice than in WT controls (Figure 7D). Additionally, as ASC aggregate numbers increased within microglia, their corresponding volumes also increased. To further analyze ASC aggregation, we categorized ASC aggregates by volume (1–20 μm^3^, 20–30 μm^3^, 30–40 μm^3^) and quantified the number of ensembles in each category (Figure 7E and Supplemental Figure 5, E and F). ASC aggregates within a range of 30–40 μm^3^ were significantly decreased in the *Nlrp3^–/–^* mice at 21 dpi (Figure 7E). Collectively, our findings demonstrate a remarkable upregulation of ASC aggregates outside of Iba1^+^ and GFAP^+^ cells, along with significant ASC aggregation at 21 dpi. *Nlrp3^–/–^* mice showed reduced ASC aggregation in Iba1^+^ cells and fewer extra-glial ASC aggregates compared with the WT group at 21 dpi. These observations highlight the essential role of NLRP3, not only in promoting inflammasome activation after brain injury but also in facilitating ASC aggregation, as previously suggested in in vitro studies (47).

### Interaction between reactive astrocytes and microglia correlates with ASC distribution following CHI.

To further investigate the relationship between reactive astrocytes and microglia interactions and ASC distribution following CHI, we conducted analysis of the correlation between ASC aggregates and glial contact areas after 3D reconstruction (Figure 7, F–I). Notably, in WT animals after CHI, we observed a strong positive correlation between the cell contact area and the number of extra-glial ASC aggregates (*r* = 0.86, *P* < 0.0001) (Figure 7G) as well as ASC aggregates in GFAP^+^ cells (*r* = 0.93, *P* < 0.0001) (Figure 7I). Although lower correlation coefficients were found between the reactive astrocytes and microglia contact areas and the number of ASC aggregates in microglia (*r* = 0.73, *P* < 0.0001) (Figure 7H) and the overall count of ASC aggregates (*r* = 0.82, *P* < 0.0001) (Figure 7F), these correlations remained significantly positive (Figure 7, F–I). Interestingly, the genetic deficiency of *Nlrp3* significantly reduced the correlation between the contact area and the number of extra-glial ASC aggregates (*z* = 1.95, *P* < 0.05) (Figure 7G) as well as ASC in GFAP^+^ cells (*z* = 2.54, *P* < 0.01) (Figure 7I). In summary, these results indicate that post-CHI ASC aggregation was partly dependent on NLRP3 and that reactive astrocytes and microglia contacts were influenced by ASC expression, distribution, and aggregation, especially the ASC aggregates located outside of Iba1^+^ and GFAP^+^ cells.

## Discussion

In this study, we identify *Asc* as a key gene expressed in monocytes, macrophages, and especially in microglia populations during the subacute phase of CHI, based on scRNA-seq data from mouse cortical cells after mFPI. Sustained ASC protein expression in our mCHI model was observed predominantly in Iba1^+^ cells (presumably microglia) and, to a lesser extent, in GFAP^+^ cells (likely reactive astrocytes). We demonstrated that *Asc^–/–^* mice exhibited attenuated neuroinflammatory responses and improved cognitive performance after mTBI, indicating ASC-dependent regulation of microglia and astrocyte reactivity and innate immune responses that contributed to neurobehavioral deficits following mTBI. Our findings not only complement and expand previous preclinical and clinical studies on moderate-to-severe TBI models (48, 49), which highlight the role of inflammasome cascades and their critical involvement in regulating secondary inflammatory responses after TBI, but also demonstrate that the main inflammasome adaptor protein ASC is a key element in neuroinflammatory responses and cognitive impairment following mTBI. In contrast to several studies on moderate-to-severe TBI models, in which the expression of inflammasome-related proteins typically peaks within the initial week following injury and declines over time (28, 50, 51), we found a progressive and sustained increase in inflammasome-related proteins, especially after the first 7 days following mCHI. This temporal pattern aligns with our observations of sustained morphological changes of reactive astrocytes and microglia during a later phase (21 dpi). These discrepancies across experimental models and TBI severities indicate that inflammasome activation within innate immune responses and tissue repair processes depends on the extent of the injury. Furthermore, the sustained proinflammatory profiles of activated microglia and reactive astrocytes imply that both glial cell types contribute to the propagation of inflammation through inflammasome-associated pathways after injury. In our study, we observed that, compared with injured WT mice, the number of Iba1^+^ cells remained stable in *Asc^–/–^* mice at both 7 and 21 dpi, indicating attenuated microglial and macrophage turnover following trauma (Figure 3, C and I). WT mice, in contrast, exhibited a distinct peak of Iba1^+^ cell proliferation and activation at 7 dpi, concurrent with the subacute neuroinflammatory response characteristic of the CHI model (42). By 21 dpi, Iba1^+^ cell density in WT mice declined toward baseline, likely reflecting ASC-dependent, CASP1-mediated pyroptotic cell death and subsequent release of proinflammatory mediators, including IL-1β and extracellular ASC aggregates. Following this microglia peak, astrocytes displayed prominent reactive morphology at 21 dpi relative to that seen at 7 dpi, possibly driven by the continued activation of neurotoxic astrocytes through microglial inflammasome signaling in response to ongoing tissue damage, as previously described in stress paradigms (52). Furthermore, these findings align with existing evidence suggesting that distinct glial cell types respond differently to brain injury in a temporal sequence (42, 53–55). Future studies should address the contribution of ASC-mediated neuroinflammation during the chronic phases of TBI (beyond 30 dpi). Notably, a recent study using a unilateral closed cortical impact model reported that Iba1 and GFAP immunoreactivity peaked at 21 dpi. While Iba1 immunoreactivity returned to baseline by 40 dpi, GFAP reactivity persisted, suggesting a longer astrocytic response throughout the chronic phase (56). Previous studies have reported that CHI elicits astrocyte reactivity through JAK/STAT3- and NF-κB–dependent signaling pathways, leading to cytoskeletal remodeling, GFAP induction, and cytokine release that support both repair and inflammation during the acute and subacute phases of injury (55, 57). Moreover, astrocytes can respond to microglia-derived factors such as IL-1α, TNF-α, and C1q, which promote distinct reactive phenotypes with predominantly proinflammatory features (43). In the chronic phase, proliferating reactive astrocytes from perilesional regions could migrate toward the lesion core to contribute to glial scar formation and wound processes. This scarring response may help contain inflammation and protect surrounding tissue but can also impede axonal regeneration and neural recovery (57).

We additionally observed widespread upregulation of inflammasome components following CHI, particularly at 21 dpi, with a substantial increase in the cleaved forms of CASP1, CASP8, and IL-1β. Our results suggest the involvement of several ASC-dependent inflammasomes in the inflammatory pathways after CHI. This was evidenced by the pronounced reduction in CASP1, CASP3 and CASP8 levels and morphological changes in microglia and reactive astrocytes we observed in *Asc^–/–^* mice. Most notably, our behavioral evaluation during the late subacute phases shows that short-term spatial memory, anxiety-related behaviors (thigmotaxis), mild procedural learning deficits, and long-term memory impairments were significantly diminished in animals with genetic deficiency of the *Asc* gene (Figures 5 and 6). Furthermore, we identified a significant positive correlation between astrocyte-microglia interactions and the number of ASC aggregates. Consistent with previous work, extracellular ASC appears to facilitate the propagation of inflammatory mediators such as IL-1β and TNF-α following inflammasome activation (15, 58). These cytokines not only promote the aggregation of glia and infiltrated cells around the trauma epicenter (59, 60), but may also influence cell communication far from the lesion site through extracellular ASC. Given its prion-like properties (58), ASC can spread to neighboring glial cells and trigger secondary activation of the ASC-mediated inflammasome cascade, as previously demonstrated in Alzheimer’s disease–related amyloidosis (61, 62). Collectively, these observations support the idea of a central role of ASC in maintaining neuroinflammation and glia reactivity after CHI.

Strikingly, *Nlrp3^–/–^* mice exhibited fewer ASC aggregates outside Iba1^+^ and GFAP^+^ cells, with a significant reduction of ASC inclusions in GFAP^+^ cells (bona fide astrocytes) but not in Iba1^+^ cells (Figure 7). This finding may reflect the constitutive abundance of ASC in microglia and a cell-type–specific regulation of inflammasome activation during neuroinflammatory responses. Recent studies have emphasized distinct inflammasome repertoires in glia subtypes. Microglia predominantly engage canonical inflammasome pathways, expressing and activating multiple complexes — including NLRP1 (63), NLRP3 (46, 51, 64), NLRC4 (65), and AIM2 (66) — depending on the nature of injury and the DAMPs involved. Upon activation, microglial inflammasomes promote CASP1 cleavage and robust IL-1β secretion, intensifying secondary neuroinflammatory cascades and neuronal damage. In contrast, astrocytes express lower basal levels of inflammasome components and appear to rely predominantly on NLRP3 for inflammasome assembly and inflammatory signaling following TBI. Astrocytic NLRP3 expression can be upregulated by microglia-derived cytokines (67, 68) or extracellular ATP released from damaged cells (69), leading to moderate CASP1 activation and IL-1β/IL-18 maturation, thereby sustaining reactive gliosis. Conditional NLRP3 deletion in astrocytes attenuates CASP1 activation and depression-like behavior after mTBI, supporting a cell-type–specific dependence on NLRP3 in astrocytes (70). Accordingly, the reduced ASC aggregation observed in astrocytes of *Nlrp3^−/−^* mice likely reflects their predominant reliance on NLRP3 for inflammasome assembly. In contrast, microglia can engage alternative inflammasome pathways (e.g., NLRP1, NLRC4, AIM2) that mediate ASC speck formation independently of NLRP3. Alternatively, diminished NLRP3 activity in microglia may reduce extracellular ASC aggregates release and intercellular propagation, further limiting secondary glial activation.

Our findings, however, contrast with a previous report on moderate CCI, in which *Asc^–/–^* mice showed no differences in motor recovery or volume lesions (30). This inconsistency may suggest that, in the context of low-grade chronic neuroinflammation induced by mild, uncontrolled stimuli such as a single concussion event, the expression, oligomerization, and propagation of ASC may occur gradually compared with the high-grade neuroinflammation triggered by severe injuries. Similarly, ASC-dependent inflammasome activation and pathological protein aggregation have been observed in several neurodegenerative disorders characterized by sustained, chronic low-grade neuroinflammation (71), such as Alzheimer’s and Parkinson’s disease, in which ASC plays a decisive role in the aggregating and further propagating amyloid β (61) and α-synuclein (72). Considering our findings, ASC may participate in immune mechanisms that potentially predispose to neurodegenerative processes following TBI (73). Nonetheless, future investigations are still required to establish a comprehensive understanding of the specific temporal participation of inflammasomes across varying injury severities and risk factors for neurodegeneration.

There are several limitations that warrant consideration in our study. While we focused on the expression of inflammasome mediators and associated cellular morphology, these parameters may not fully reflect the intricate interplay between compensatory mechanisms operating within affected neuronal networks or the broader antiinflammatory modulation of secondary immune responses. Furthermore, although our scRNA-seq analysis identified specific cellular clusters upregulating *Asc* during the subacute phase of the CHI model, the use of a conventional global KO line limited our ability to delineate the distinct contributions of peripheral and central cell populations to the post-injury immune response.

Future research should aim to delineate the longitudinal neuroinflammatory trajectory across defined neural, glial, and peripheral cellular compartments, particularly using inducible KO models and pharmacological interventions targeting ASC aggregation or inflammasome assembly. In this context, recently developed orally bioavailable NLRP3 inhibitors have shown beneficial effects on neurological recovery, ASC aggregation, and microglia morphology after moderate CCI (46). Moreover, humanized monoclonal antibodies such as IC100 (74) and single-domain antibodies targeting ASC (75) have demonstrated preclinical efficacy in other inflammatory conditions, supporting their potential translational relevance to target ASC and ASC-dependent inflammasome signaling in clinical trials of TBI.

Our findings pave the way to delve into specific pharmacological agents that can effectively modulate ASC expression, activity and aggregation and assess their effect on neurological outcomes after TBI. Considering our findings, pharmacological interventions targeting ASC may help to mitigate neuroinflammation and potentially promote neuroprotection, enhancing the recovery of injury and preventing further neurodegeneration and disability.

## Methods

### Sex as a biological variable.

Sex was not considered as a biological variable in this study.

### Mice.

Male and female mice (approximately 50% of each sex), aged 6–7 months, on a mixed C57BL/6 background (C57BL/6N-J), were used in all experiments. All animals were bred and housed in the House of Experimental Medicine at the University Hospital Bonn in Germany. WT, *Nlrp3^–/–^*, and *Asc^–/–^* mice were initially sourced from Millennium Pharmaceuticals as previously described (76). All mice were backcrossed with C57BL/6N mice and maintained under pathogen-free, standardized conditions with a 12-hour light/12-hour dark cycle and ad libitum access to food and water. Behavioral experiments were conducted during the dark phase of the light/dark cycle.

### Electromagnetic controlled CHI model of mTBI.

To model mTBI in mice, we adapted the previously described electromagnetic controlled closed-head model (77) (Supplemental Figure 2A). Briefly, mice were anesthetized with 5% isoflurane during induction, and 1.5%–2% isoflurane mixed with 100% oxygen (0.5–1 L/min) during surgery and then fixed in a stereotaxic frame (Stoelting). A 1 mL latex pipette bulb filled with water was placed under the head to distribute the impact force. A midline sagittal scalp incision was made, and a single, controlled midline skull impact was delivered at coordinates 0.0 mm mediolateral and –1.5 mm anteroposterior, with a velocity of 5 m/s, a dwell time of 100 ms, and an impact depth of 1.0 mm, using a stereotaxic electromagnetic impactor with a 5.0 mm steel tip (Stereotaxic Impactor, Leica Biosystems). Sham-treated mice underwent identical surgical procedures without impact injury. In the postoperative phase, mice received carprofen (5 mg/kg) s.c. once daily and tramadol (1 mg/mL) in the drinking water for 3 days.

### NSS-R.

The NSS-R consists of a structured series of neurological tests evaluating motor and sensory abilities, including assessments of general mobility, reflex inhibition, and postural control (36). Scores range from 0, indicating normal function, to 20, representing the most severe impairment.

### Rotarod test.

Motor coordination and balance were assessed using an accelerating rotarod apparatus (Ugo-Basile). The rotation speed progressively increased from 5 to 40 revolutions per minute over a 5-minute testing period. Mice underwent 3 trials daily for 4 consecutive days prior to CHI, followed by 2 additional testing sessions after the injury. The latency to fall and the rotational speed at the time of the fall were automatically recorded by the system for each trial.

### NBT.

The NBT was used to assess natural, species-typical behaviors indicative of daily functional capacities. Individual mice were housed alone and provided standardized nesting material in their home cages at the beginning of their active dark cycle. Mice were given 12 hours to construct nests, after which nest quality was evaluated. Nest scoring was done using a validated scale from 1–5, with consideration of factors such as structure, coherence, and completeness of the nests, thereby quantifying functional nesting performance (78).

### EOM.

Anxiety-related behavior and risk evaluation were measured using an EOM apparatus composed of a circular elevated runway (46 cm diameter, 5.5 cm width) positioned 40 cm above the ground. The maze featured 2 enclosed arms alternating with 2 open sectors.

### OF test.

Voluntary locomotion and exploratory activity were examined in a 50 cm cubic OF chamber constructed from opaque gray acrylic. Mice were recorded for 15 minutes per session, under low indirect lighting conditions (~40 lux).

### Novel OLM test.

The novel OLM test was performed within the OF arena with visual cues on one of the walls, with the floor covered in a 1 cm layer of used and smell-saturated bedding material. Following a 1-day habituation period in which mice explored the arena with 3 identical colored Lego objects (2 × 4 cm) placed in fixed positions for 5 minutes daily, the test day began with a 6-minute exploration of these objects. The object investigation time was tracked automatically by Noldus EthoVision software. After a 1-hour delay, 1 object was relocated to a new position, while the other 2 objects remained stationary. The animals were reintroduced for a 5-minute session to assess their preference for the displaced object. The discrimination index (percentage) was calculated during the initial 3 minutes as follows: (time exploring relocated object/total exploration time of all objects) ×100.

### MWM.

Spatial learning and memory evaluation was conducted between 10 and 20 dpi. A circular pool with a diameter of 1 m was filled with white opacified water maintained at a temperature of 21°C–23°C. The pool was dimly lit (approximately 40 lx) and surrounded by a white curtain. Asymmetrically placed distal cues were positioned on the pool wall to serve as spatial references. The pool was virtually divided into 4 quadrants, 1 of which contained a hidden platform (15 cm in diameter) submerged 1.5 cm below the water’s surface. Mice were trained to locate the platform using the distal cues for orientation. Training consisted of 4 trials per day for 5 consecutive days. During each trial, the mice were placed in the water, facing the pool wall in a quasi-randomized order to prevent them from developing fixed strategies. Mice were given 60 seconds to find the platform; if they failed to locate it within this time, they were manually guided to it. Once on the platform, the mice were allowed to remain there for 15 seconds before the next trial began. After completing all 4 trials for the day, the mice were dried and returned to their home cages. The integrated time or distance traveled during these trials was analyzed. A spatial probe trial was conducted 24 hours after the final training session (16 dpi). For this test, the hidden platform was removed, and the mice were allowed to swim freely for 60 seconds. The drop position was in the quadrant opposite of where the platform had been located, with each mouse facing the wall at the center of that quadrant. One day after the spatial probe trial, a visually cued learning phase began. During this phase, the platform was flagged for visibility and relocated to a different quadrant, while all distal cues were removed. Mice underwent 3 trials per day for 3 consecutive days (17–20 dpi) to learn to locate the flagged platform. All mouse movements were recorded and tracked using Noldus EthoVision software.

### Tissue preparation.

Mice were deeply anesthetized using ketamine (100 mg/kg) and xylazine (20 mg/kg) and transcardially perfused with at least 30 mL ice-cold PBS at designated time points (sham, 1, 7, and 21 dpi). Following perfusion, the brains were carefully dissected. One hemisphere was snap-frozen in liquid nitrogen and stored at –80°C until biochemical analyses, while the other hemisphere was fixed in 4% paraformaldehyde overnight. Coronal brain sections (40 μm thick) were obtained using a Leica VT1000S vibratome (Leica Microsystems) from the region spanning bregma –0.45 to –1.85 mm. Sections were collected in PBS at intervals of 300 μm for various staining procedures.

### Primary antibodies.

For immunoblotting, the following primary antibodies were used: mouse anti-CASP1 (1:1,000; AdipoGen; AG-20B-0042); rabbit anti-ASC (1:1,500; Cell Signaling Technology; D2W8U); rabbit anti-NLRP3 (1:500; Cell Signaling Technology; D4D8T); rabbit anti–IL-1β from (1:1,000; E7V2A; Cell Signaling Technology); rabbit anti-CASP3 (1:1,000; D3E9; Cell Signaling Technology); and rabbit anti-CASP8 (1:1,000; D5B2; Cell Signaling Technology). For immunohistochemical analysis, the antibodies included rabbit anti-Iba1 (1:1,000; Fujifilm Wako reagents, 019-19741); goat anti-Iba1 (1:1,000; Abcam; EPR16588); rat anti-GFAP (1:300; Thermo Fisher Scientific; 2.2B10); goat anti–IL-1β (1:500; R&D Systems; AF-401-SP), rabbit anti-ASC (1:1,500; Cell Signaling Technology; D2W8U); and rabbit anti-CASP3 (1:1,000; D3E9; Cell Signaling Technology).

### Tissue protein extraction and immunoblotting.

After thawing, brain tissue was homogenized in PBS containing 1 mM EDTA, 1 mM EGTA, and the protease inhibitor cocktail Halt (Thermo Fisher Scientific). The homogenates were extracted in RIPA buffer (25 mM Tris–HCl [pH 7.5], 150 mM NaCl, 1% Nonidet P-40, 0.5% NaDOC, 0.1% SDS) and centrifuged at 20,000*g* for 30 minutes. RIPA fractions were separated using NuPAGE electrophoresis gels (Thermo Fisher Scientific) and immunoblotted with primary antibodies, followed by incubation with the corresponding secondary antibodies. Immunoreactivity was detected with an Odyssey CLx Imaging System (LI-COR), and images were analyzed using ImageJ (NIH).

### ELISA proinflammatory cytokine panel.

IL-1β and TNF-α levels in RIPA fractions were measured using the V-PLEX Plus Mouse ProInflammatory Panel 1 (Meso Scale Discovery), following the manufacturer’s protocol. Samples were diluted 1:1 on the plate with the reagent diluent provided in the kit. Signals were detected using a QuickPlex SQ 120 plate reader (Meso Scale Discovery).

### IHC.

Brain sections were washed 3 times for 5 minutes with PBS and incubated in citrate buffer for 5 minutes at 95°C. After antigen retrieval, the sections were cooled down at room temperature and washed with PBS and in 0.5% Triton X-100 B (PBST), and then blocked for 1 hour with 1% BSA in PBST and incubated overnight with the primary antibodies. Next, the sections were washed 3 times for 5 minutes in PBST, incubated with appropriate secondary antibodies (1:1,000) for 60 minutes, followed by 3 washes with PBS washing for 5 minutes. The tissue was mounted using ProLong Gold Antifade Mountant with DNA Stain DAPI (Thermo Fisher Scientific). Three coronal brain sections per animal (between bregma –1.00 mm and –1.85 mm) were selected. Confocal *Z*-stack images of the contusion were primarily acquired from layers 1 and 2/3 of the retrosplenial and primary motor cortex. Each *Z*-stack consisted of 10 optical slices (*c* × *z* × *t* = 10), with 1 frame captured per acquisition. Imaging parameters were standardized across all samples: the pixel size was set to 0.1559814 μm in both the *x* and *y* axes, and the voxel depth (*z*-step) was maintained at 2.0 μm.

### Skeleton analysis of microglia and reactive astrocytes.

Morphological changes in microglia and reactive astrocytes were quantitatively analyzed using a skeleton analysis method (79). Images were imported into ImageJ software, converted to grayscale, and adjusted for brightness and contrast to enhance cell process visibility. After obtaining binary images of microglia and reactive astrocytes, we used the plugin skeletonize to obtain skeletons, representing the medial axis of cell processes. Morphological parameters measured included process branches, defined as the number of branching points; endpoints, defined as the number of terminal points; and process lengths, defined as the total length of all processes. The data were normalized by dividing the measurements by the number of cells in each image.

### Contact analysis between microglia and reactive astrocytes.

Imaris Image Analysis Software (version 9.5, Oxford Instruments) was used to analyze the contact area between microglial and astrocyte surfaces. 3D reconstructions were created using the tool surfaces by setting intensity thresholds to differentiate cell surfaces from the background. Adjustments were made to ensure accurate cell boundary representation. The Surface-Surface Contact X-Tension identified and analyzed overlapping regions between microglia and astrocyte surfaces, generating a new surface to represent the contact area. The contact area (μm^2^) was calculated on the basis of the number of overlapping voxels within the 3D reconstructions.

### Analysis of ASC aggregates.

The cortical impact area was examined using a confocal LSM900 microscope (Zeiss). Images were captured under consistent confocal settings and processed with Imaris Image Analysis Software (Oxford Instruments) to identify ASC aggregates. Standardized settings were maintained across samples, including pinhole size, laser intensity, digital gain, and offset. *Z*-stacks were collected to obtain the complete 3D structure of cells and ASC aggregates. Image preprocessing included adjusting brightness and contrast, reducing noise, and subtracting the background to enhance aggregate visibility. Imaris Image Analysis Software was used to create 3D reconstructions of microglia, astrocytes, and ASC aggregates. The “Surfaces” tool generated accurate 3D models by setting intensity thresholds. The “Spots” tool identified and quantified ASC aggregates on the basis of fluorescence intensity and size, with thresholds set using positive (mCherry-ASC mice) and negative (*Asc^–/–^* mice) controls. The “Spots Close to Surface” tool analyzed the spatial distribution of ASC aggregates relative to microglia or astrocytes, indicating potential inflammasome activation.

### IL-1β analysis.

Coimmunostainings for IL-1β, Iba1, and GFAP were performed at 7 and 21 dpi. 3D reconstructions of microglia, reactive astrocytes, neuronal nuclei, and IL-1β signals were generated using Imaris Image Analysis Software (Oxford Instruments). The “Spots” tool was used to quantify IL-1β signals in the 3D space by analyzing fluorescence intensity and particle size thresholds. To assess spatial relationships, the “Spots Close to Surface” tool measured the proximity of IL-1β signals to cellular surfaces, evaluating potential associations with microglia, astrocytes, or neurons. Quantitative metrics, including the number of IL-1β spots adjacent to specific cell types, were measured for statistical analysis.

### scRNA-seq data analysis.

Raw FASTQ files and count matrices are publicly available in the NCBI Gene Expression Omnibus (GEO) database under accession number GSE160763 (31). scRNA-seq data were analyzed using Seurat, version 5.1.0 in R, version 4.3.2 (80). Briefly, cells with over 5,000 detected genes, unique molecular identifier counts under 12,500, and less than 1% mitochondrial gene content were retained, followed by log transformation of the data. We selected the top 4,000 highly variable genes for principal component analysis (PCA) and used the top 20 principal components as input for UMAP dimensionality reduction. Cell-cycle states were determined using Seurat’s CellCycleScoring (81) function with marker genes from Tirosh et al. (80). Batch correction was performed using Harmony to integrate datasets across samples (82). Clusters were identified at a resolution of 0.5. Cellular identities were assigned via automated cell-type annotation using SingleR (34), which assigns cellular identity for single-cell transcriptomes by comparison with reference data sets of pure cell types sequenced by microarray or RNA-seq. Annotation relied on a reference dataset of 358 mouse RNA-seq samples annotated to 18 major cell types (83). Figures were generated using Tidyverse (84), SCpubr (85), and Enhanced Volcano (86).

### Statistics and reproducibility.

Data other than scRNA-seq were analyzed using GraphPad Prism Software version 9.0 or later (GraphPad Software) and are presented as the mean ± SEM. Immunohistochemical quantifications and behavioral analyses were performed by investigators blinded to the genotype and experimental condition. After assessing the distribution, statistical comparisons were performed using either a 2-tailed *t* test or 1- or 2-way ANOVA with Bonferroni’s or Tukey’s post hoc test to identify group differences. Morris Water Maze (MWM) learning data were analyzed using repeated-measures, 2-way ANOVA with Tukey’s post hoc test. Correlation analyses were performed using nonparametric Spearman’s correlations. Differences between Spearman’s correlation coefficients were assessed using the standard Fisher’s *z*-transformation and subsequent comparison (87). Results were considered statistically significant at a *P* value of less than 0.05.

### Study approval.

We followed the European guidelines for animal research, conformed to the requirements of the German Animal Welfare Act, and received approval from North Rhine-Westphalia State Agency for Nature, Environment and Consumer Protection under folder number 81-02.04.2019.A026 and 2024-791-Grundantrag.

### Data availability.

Values for all data points in graphs are reported in the Supporting Data Values file. Sequencing analysis was performed on previously published, publicly available data (31). Raw FASTQ files and count matrices are available in the NCBI GEO database (GSE160763).

### Code availability.

Code used to an analyze the scRNA-seq data is available at https://gitlab.com/uniluxembourg/lcsb/neuroinflammation/rna_single_seq

## Author contributions

SCG and MTH conceptualized the study. AVS, PBL, SCG, TL, SS, Y Ding, and MG designed the study methodology. AVS, PBL, SCG, TL, SS, Y Ding, and Y Deng performed experiments. SCG and MTH acquired funding. SCG and MTH supervised the study. SCG, TL, and Y Deng wrote the original draft of the manuscript. MTH, SCG, PB, TL, Y Ding, Y Deng, DTG, VS, and EL wrote and edited the manuscript.

## Funding support

- Alzheimer Forschung Initiative e.V (grant 21060, to SCG)
- Hertie Network of Excellence in Clinical Neuroscience (grant 2021-1A-12, to SCG).
- Deutsche Forschungsgemeinschaft (DFG) – Neuro-aCSis, Bonn Neuroscience Clinician Scientist Program (project 493623632, to SCG).
- DFG – ImmunoSensation2 (EXC2151–390873048, to SCG and MTH).
- National Strategy for Translational Projects for Gene and Cell Therapy Products and Associated Diagnostics from the Federal Ministry of Education and Research (BMBF) (grant 01_BIH_TP_2410, to SCG).
- Fonds National de la Recherche (FNR) within the PEARL program (FNR/16745220, to MTH and PBL).
- China Scholarship Council (CSC) (file no. 202308310126, to Y. Ding and file no. 201908510162, to Y. Deng).
- Academic Program Department of Biotherapy Cancer Center and State Key Laboratory of Biotherapy, West China Hospital, Sichuan University (to TL and MG).

## Supplementary Material

Supplemental data

Unedited blot and gel images

Supporting data values

## Version 1 02/24/2026

In-Press Preview

## Version 2 04/01/2026

Electronic publication
