# Supplementary material for: Inflammasome adaptor ASC promotes sustained neuroinflammation and mild cognitive impairment in a closed-head injury model
Source: J Clin Invest. 2026 Feb 24;136(7):e199818. doi: 10.1172/JCI199818 (PMC13038205; doi:10.1172/JCI199818)
Supplement: Supplemental data [file jci-136-199818-s208.pdf]

Supplemental data

Inflammasome adaptor ASC promotes sustained neuroinflammation and mild cognitive impairment in a closed-head injury model

Tao Li*[1], Sergio Castro-Gomez*#[1,2], Pablo Botella Lucena[3], Ana Vieira-Saecker[1], Stephanie Schwartz[1,2], Yingying Ding[2], Yushuang Deng[4], Maling Gou[5], Valentin Stein[2], Douglas T. Golenbock[6], Eicke Latz[7,8,9], Michael T. Heneka#[3,6]

1. Clinic of Parkinson, Sleep and Movement Disorders, Center for Neurology, University Hospital Bonn, University of Bonn, Bonn, Germany
2. Institute of Physiology II, University Hospital Bonn, University of Bonn, Bonn, Germany
3. Luxembourg Centre for Systems Biomedicine (LCSB), University of Luxembourg, Belvaux, Luxembourg
4. German Center for Neurodegenerative Diseases (DZNE), Bonn, Germany
5. Department of Biotherapy, Cancer Center and State Key Laboratory of Biotherapy, West China Hospital, Sichuan University, Chengdu, PR China
6. Division of Infectious Diseases and Immunology, University of Massachusetts Medical School, Worcester, USA
7. Institute of Innate Immunity, University Hospital Bonn, Bonn, Germany
8. Centre of Molecular Inflammation Research, Norwegian University of Science and Technology, Trondheim, Norway
9. Deutsches Rheuma-Forschungszentrum (DRFZ), Berlin, Germany

***Authorship notes:** TL and SC-G contributed equally to this work.

#**Correspondence**: michael.heneka@uni.lu, sergio.castro-gomez@ukbonn.de

Competing interests: M.T.H. is a scientific advisory board member at Alector the Dementia Discovery Fund, and Muna Therapeutics and has received honoraria for oral presentations from Pfizer, Novartis, Roche, Abbvie, and Biogen. E.L. is a co-founder and adviser at IFM Therapeutics, Dioscure Therapeutics, Stealth' Biotech, and Odyssey Therapeutics.

Supplemental Figure 1: Single-Cell Transcriptomic Analysis of Brain Immune Cells Following TBI.

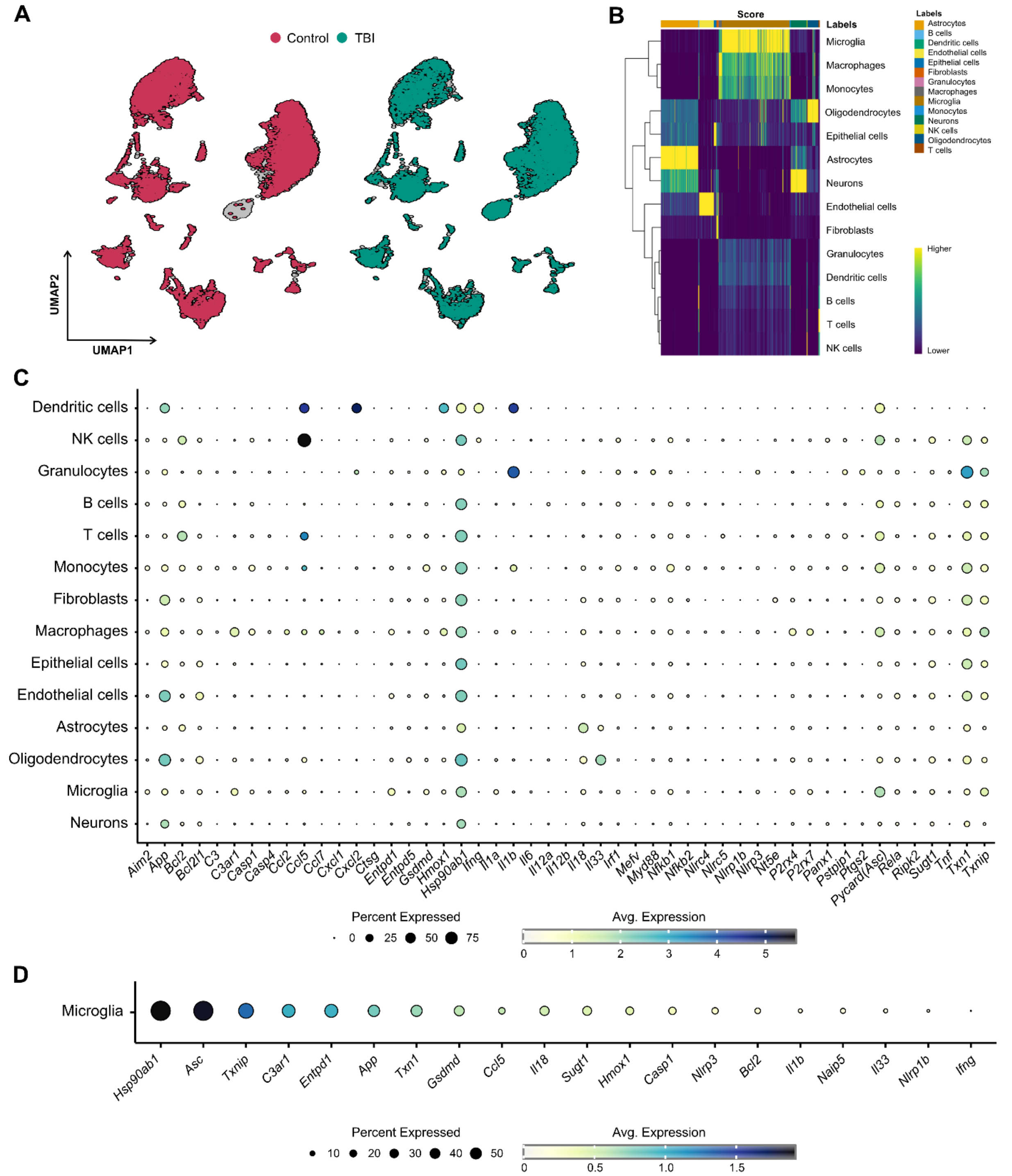

A. UMAP clustering of single-cell RNA sequencing (scRNA-seq) data, comparing control (red) and TBI (green) samples, illustrating shifts in immune cell populations post-TBI. **B.** Heatmap showing gene expression profiles across different brain cell types and cluster identification. **C.** Dot plot depicting the

expression of key inflammasome-related genes across different brain cell types. **D.** Dot plot depicting gene expression profile of inflammasome-related genes in microglia after TBI.

Supplemental Figure 2: ASC modulates expression and cleavage of CASP-8 and CASP-3 following CHI.

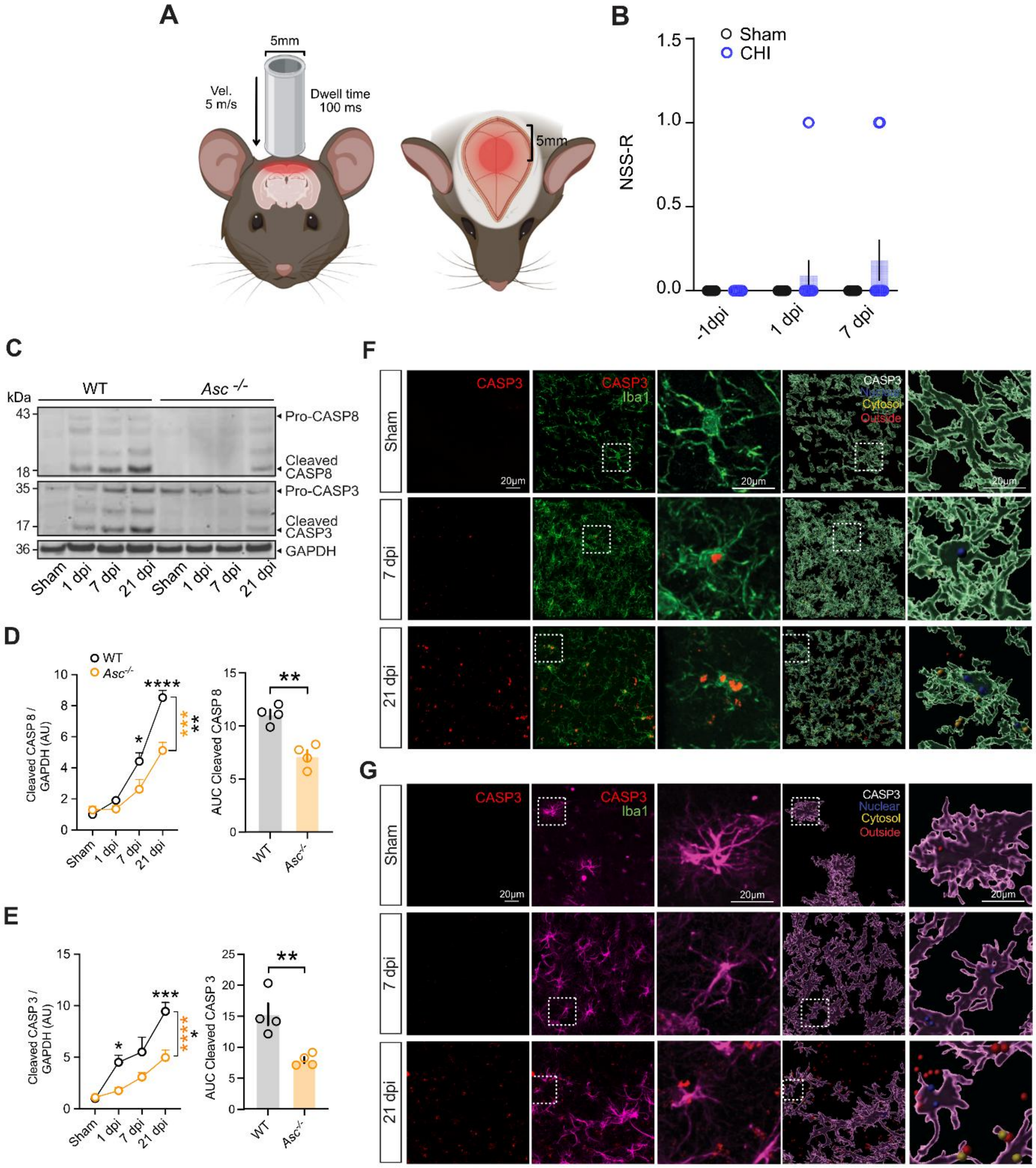

A. Schematic of injury model/location. Coronal view (right) shows tip positioned over the skull midline. Horizontal view (left) shows impact site relative to bregma. **B.** No significant motor, reflex or reaction deficits at 1- or 7-days post-intervention (dpi) when the mice were evaluated using the Revised Neurobehavioral Severity Scale (NSS-R). (Two-way ANOVA followed by Bonferroni's post-hoc tests, n = 11 mice per group per each time point, data are presented as the mean ± SEM)**. C.** Representative immunoblot images of

CASP 8 and CASP 3 at 1, 7, and 21 dpi in peri-contusional cortices of mice subjected to Sham or CHI, comparing WT and *Asc* $^{-/-}$ groups. **D-E.** Quantification of expressions of CASP 8 and CASP 3. The band intensity of a given target protein was normalized to the corresponding GAPDH signal for each sample. Data were further normalized to the average of the corresponding WT group and are presented as the mean ± SEM. Statistical significance was determined using two-way ANOVA followed by Bonferroni's post-hoc tests, n = 4 mice per group per each time point, *p < 0.05, **p < 0.01, ***p < 0.001, ****p < 0.0001 (left panels). Quantification of area under the curve (AUC) of each target over time (dpi) in WT and $Asc^{-/-}$ mice is shown in right panels. Statistical analysis was performed using an unpaired two-tailed t-test. Data are presented as the mean ± SEM. *p < 0.05, **p < 0.01, ***p < 0.001, ****p < 0.0001. **F–G**. Colocalization analysis of CASP3 immunoreactivity with $Iba1^{+}$ and $GFAP^{+}$ cells. Left panels show 3D reconstructions; blue dots indicate colocalization with the nuclear marker DAPI, yellow dots represent cytoplasmic localization, and red dots denote localization outside the cell.

Supplemental Figure 3: The cellular source of IL-1β following mTBI.

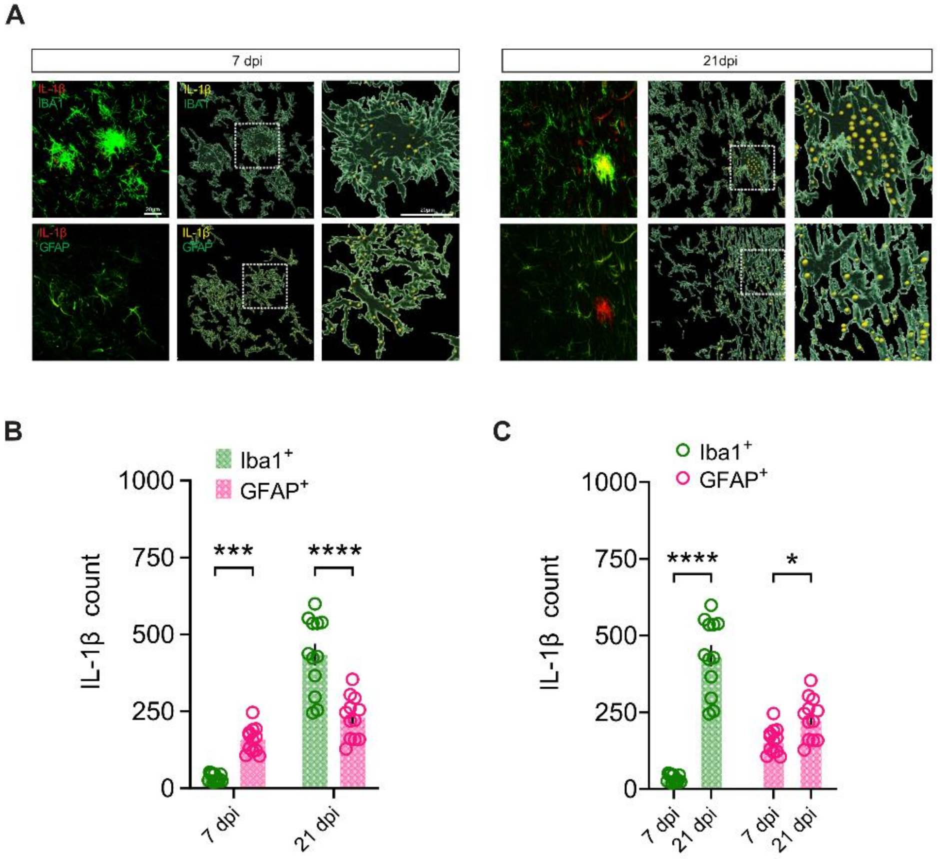

A. Representative images of immunohistochemical staining of the contusional cortices for IL-1β (red), Iba1 (green) and GFAP (green) in mice following sham surgery and 7 dpi and 21 dpi after CHI and 3D reconstruction of IL-1β immunostaining at 7 dpi and 21 dpi following CHI. IL-1β (yellow spots) localization within $Iba1^+$ and $GFAP^+$ cells. Scale bar, 20 µm. **B-C.** IL-1β spot counts assessed in $Iba1^+$ and $GFAP^+$ cells at 7 and 21 dpi following CHI. Data are presented as the mean ± SEM. Statistical significance was determined using two-way ANOVA followed by Bonferroni's post-hoc tests, n = 12 slices (4 mice) per group per each time point, *$p < 0.05$, **$p < 0.01$, ***$p < 0.001$, ****$p < 0.0001$.

Supplemental Figure 4: Mild Cognitive Impairment after CHI

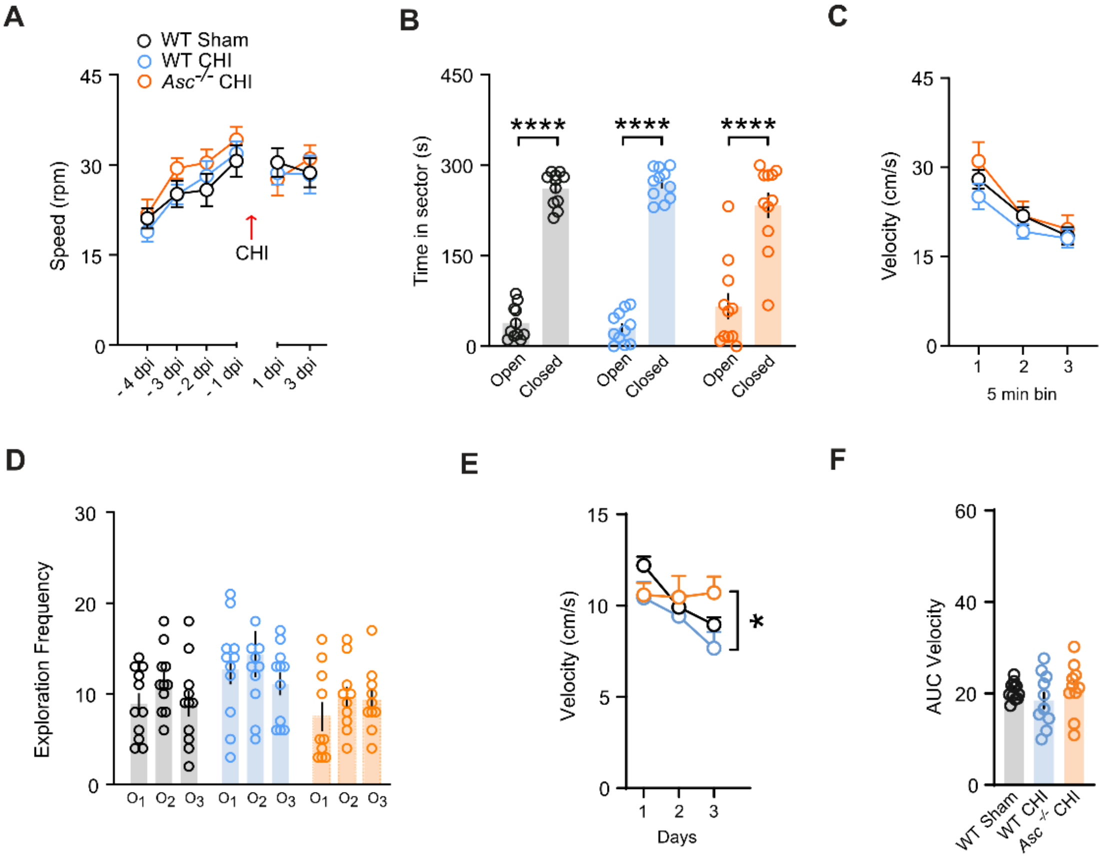

A. Rotarod performance, measured as falling speed, is comparable among WT Sham, WT CHI, and *Asc*$^{-/-}$ mice. **B**. WT Sham, WT CHI and *Asc*$^{-/-}$ mice spend significantly longer time exploring closed sectors compared to open sectors in the elevated O maze (EOM). **C**. Open field (OF) testing reveals similar locomotor velocities in WT Sham, WT CHI and *Asc*$^{-/-}$ mice. **D**. The frequency of exploration of the three identical objects is comparable across WT Sham, WT CHI and *Asc*$^{-/-}$ mice during the habituation phase of the Novel Object Location Memory test (OLM). **E**. Swimming velocity during cued learning in Sham WT, CHI-injured WT, and CHI-injured *Asc*$^{-/-}$. **F.** Comparison of the integrated area under the curve of swimming velocity. (n= 10-11 mice per genotype, *$p < 0.05$, **$p < 0.01$, ***$p < 0.001$ by ordinary one-way and two-way ANOVA with Tukey's post-hoc tests). Data are presented as mean ± SEM.

85 **Supplemental Figure 5: ASC antibody validation and ASC aggregates analysis.**

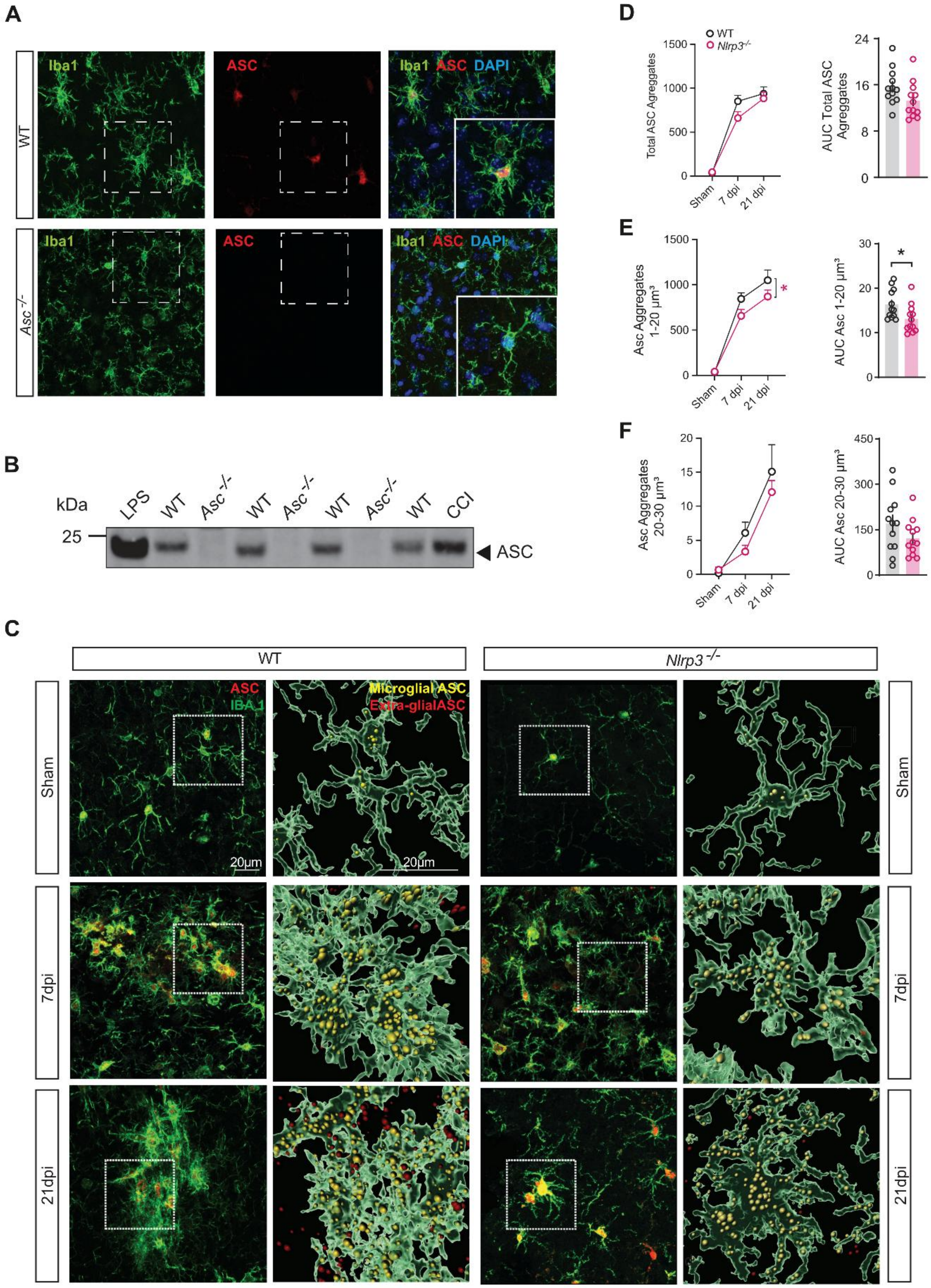

86

87

A-B. Validation of ASC antibody specificity using immunofluorescence staining and immunoblotting. **A.** ASC signal is detected in WT mice but absent in *Asc*$^{-/-}$ mice. **B.** Precipitated supernatant from LPS-stimulated primary microglia and cortical tissue from CCI brain-injured mice were used as positive controls. **C.** Representative immunohistochemical images of Iba1 (green) and ASC (red) in peri-contusional cortex of WT and *Nlrp3*$^{-/-}$ mice at different time points (sham, 7 dpi, 21 dpi). Same samples as Figure 7A. ASC aggregates were analyzed and 3D reconstructed using IMARIS. Scale bar, 20 μm. **D-F.** Quantification of ASC aggregation and ASC aggregates volume. Two-way ANOVA with Bonferroni's post-hoc tests (*p < 0.05, **p < 0.01, ***p < 0.001, ****p < 0.0001). n = 12 slices (4 mice) per group per each time point. Data are shown as mean ± SEM.
