# Supplementary material for: Inflammasome adaptor ASC promotes sustained neuroinflammation and mild cognitive impairment in a closed-head injury model
Source: J Clin Invest. 2026 Feb 24;136(7):e199818. doi: 10.1172/JCI199818 (PMC13038205; doi:10.1172/JCI199818)

Inflammasome adaptor ASC promotes sustained neuroinflammation and mild cognitive impairment in a closed-head injury model

Tao Li*[1], Sergio Castro-Gomez*#[1,2], Pablo Botella Lucena[3], Ana Vieira-Saecker[1], Stephanie Schwartz[1,2], Yingying Ding[2], Yushuang Deng[4], Maling Gou[5], Valentin Stein[2], Douglas T. Golenbock[6], Eicke Latz[7,8,9], Michael T. Heneka#[3,6]

1. Clinic of Parkinson, Sleep and Movement Disorders, Center for Neurology, University Hospital Bonn, University of Bonn, Bonn, Germany
2. Institute of Physiology II, University Hospital Bonn, University of Bonn, Bonn, Germany
3. Luxembourg Centre for Systems Biomedicine (LCSB), University of Luxembourg, Belvaux, Luxembourg
4. German Center for Neurodegenerative Diseases (DZNE), Bonn, Germany
5. Department of Biotherapy, Cancer Center and State Key Laboratory of Biotherapy, West China Hospital, Sichuan University, Chengdu, PR China
6. Division of Infectious Diseases and Immunology, University of Massachusetts Medical School, Worcester, USA
7. Institute of Innate Immunity, University Hospital Bonn, Bonn, Germany
8. Centre of Molecular Inflammation Research, Norwegian University of Science and Technology, Trondheim, Norway
9. Deutsches Rheuma-Forschungszentrum (DRFZ), Berlin, Germany

***Authorship notes:** TL and SC-G contributed equally to this work.

#**Correspondence**: michael.heneka@uni.lu, sergio.castro-gomez@ukbonn.de

Competing interests: M.T.H. is a scientific advisory board member at Alector the Dementia Discovery Fund, and Muna Therapeutics and has received honoraria for oral presentations from Pfizer, Novartis, Roche, Abbvie, and Biogen. E.L. is a co-founder and adviser at IFM Therapeutics, Dioscure Therapeutics, Stealth' Biotech, and Odyssey Therapeutics

Fig. 2A

**Anti-NLRP3**	**kDa**
**NLRP3**	**115**
**anti-GAPDH**	**40**

Blot 1

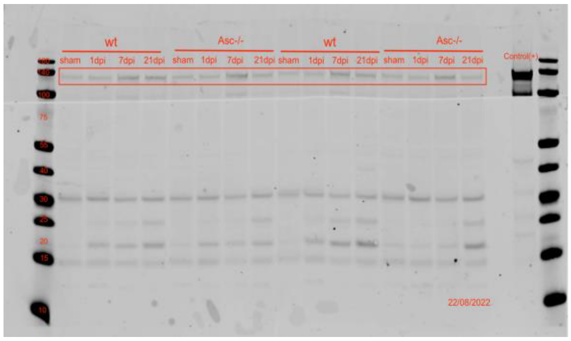

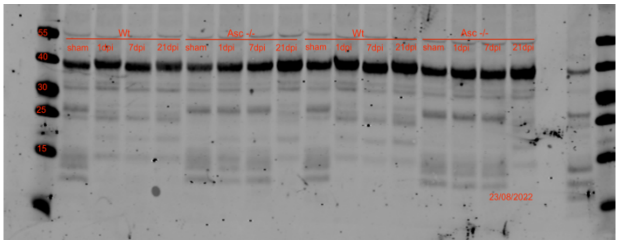

Blot 2

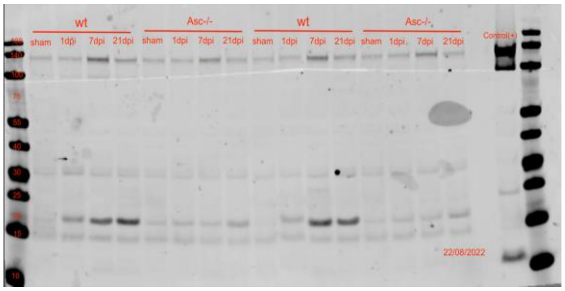

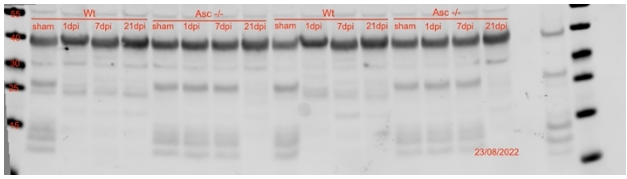

**anti-Casp1**	**Kda**	
	**Pro-Casp1**	**50**
	**Cle-Casp1**	**20**
	**anti-GAPDH**	**40**

Blot 1

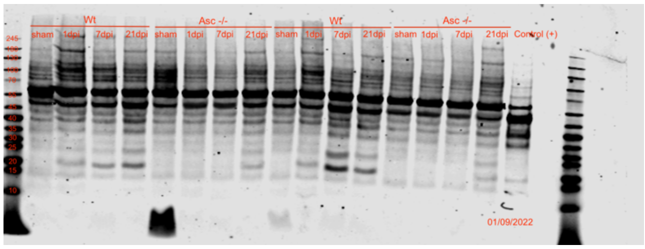

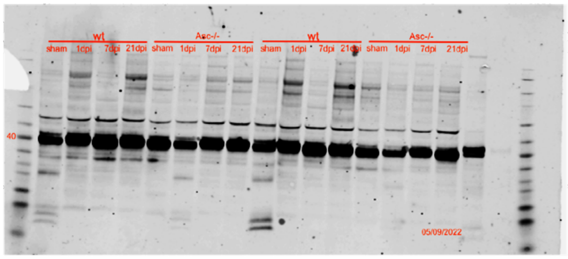

Blot 2

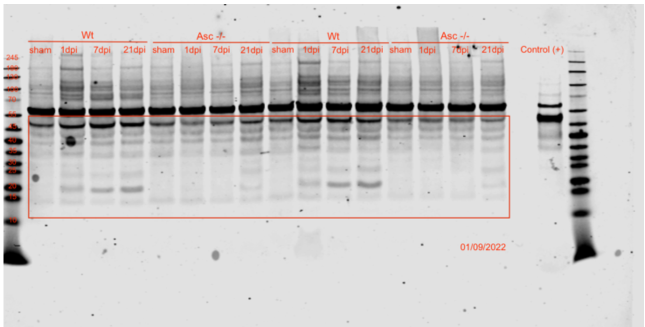

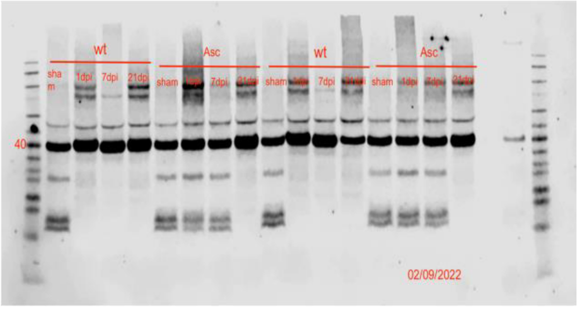

**anti-il-1b 1**	**Kda**	
	**Pro il-1b**	**30**
	**Cle il-1b**	**20**
	**anti-GAPDH**	**40**

Blot 1

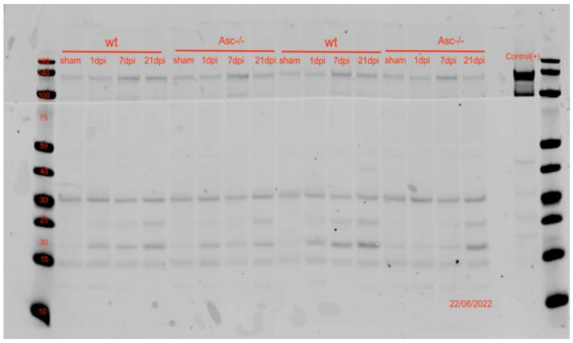

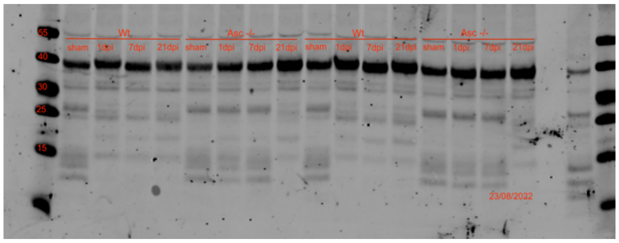

Blot 2

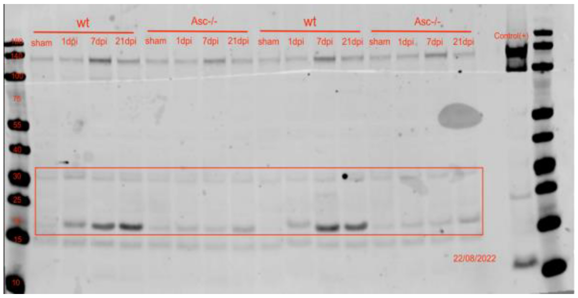

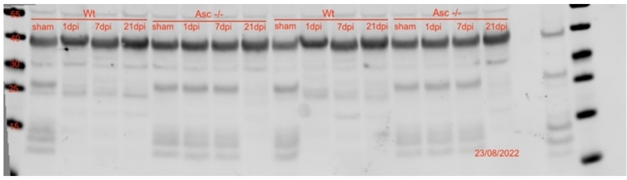

**anti ASC**	**Kda**	
	**Asc**	**25**
	**anti-GAPDH**	**40**

Blot 1

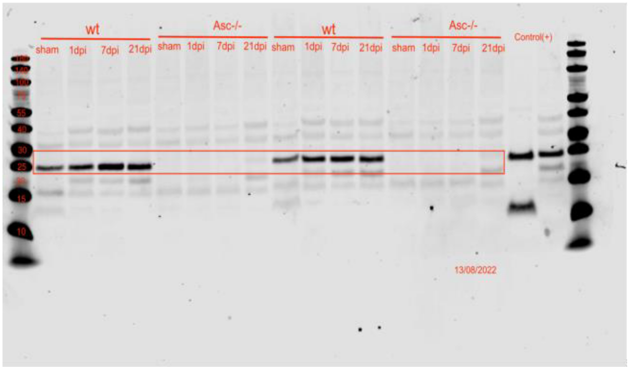

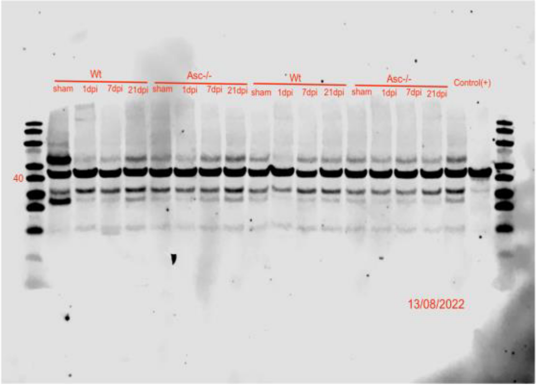

Blot 2

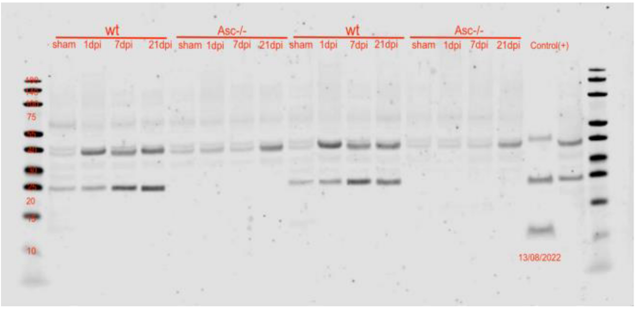

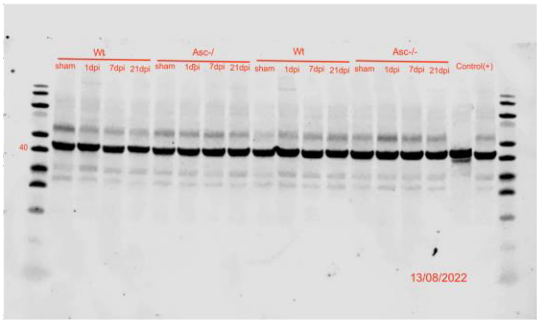

Suppl. Fig. 2C

**anti-Caspase 8**	**Kda**	
	**Caspase 8**	**43, 19**
	**anti-GAPDH**	**40**

Blot 1

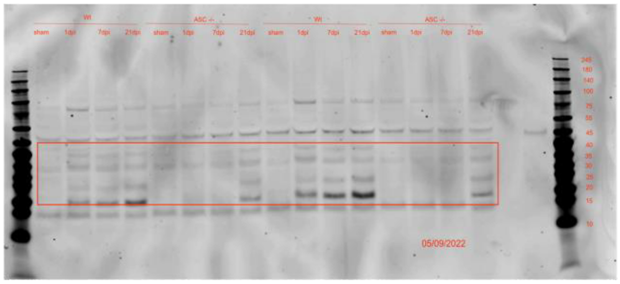

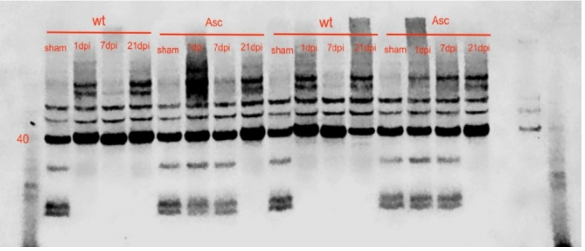

Blot 2

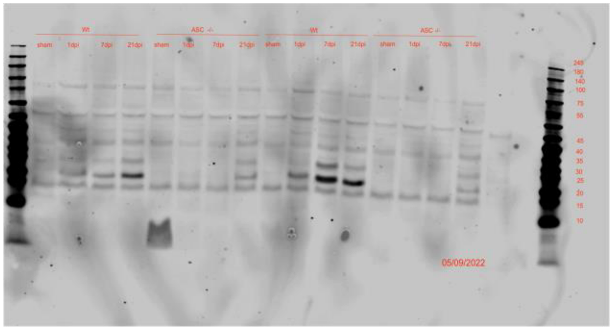

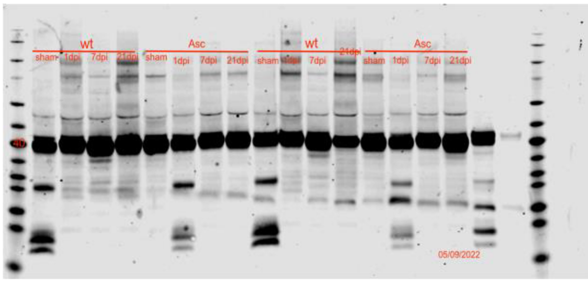

**anti-Caspase 3**	**Kda**	
	**Caspase 3**	**30, 19**
	**anti-GAPDH**	**40**

Blot 1

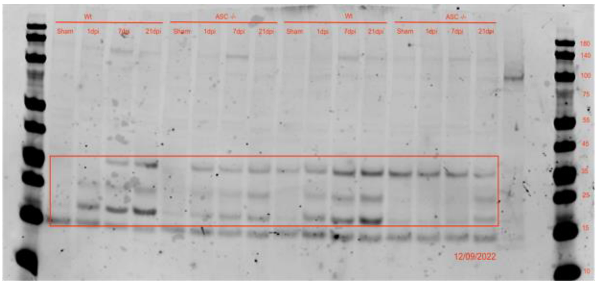

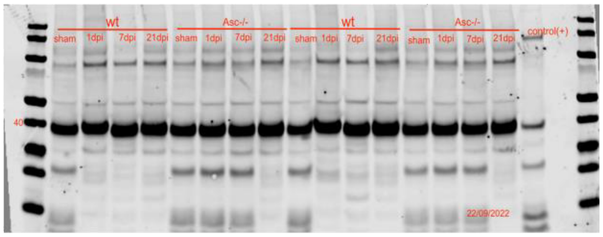

Blot 2

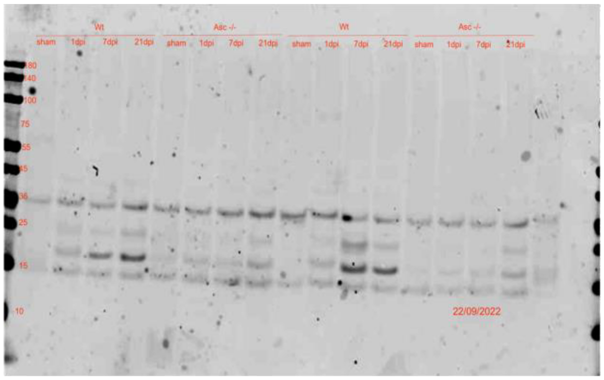

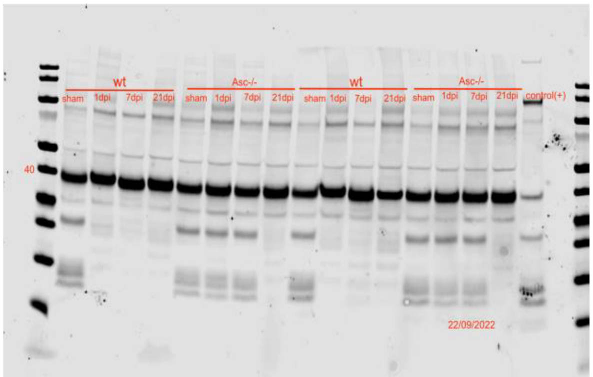

Suppl. Fig. 5B

anti ASC **Kda**

Asc **25**

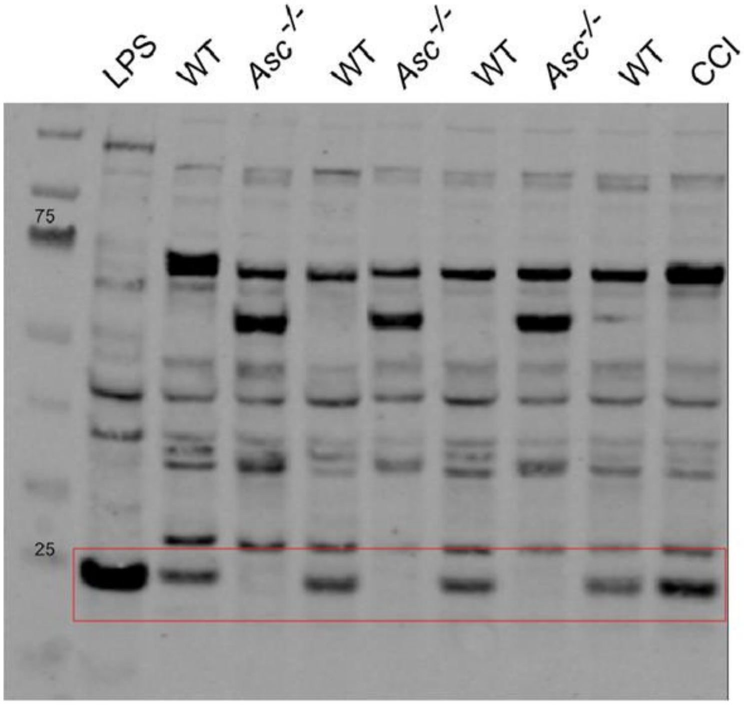

Supplement: Unedited blot and gel images [file jci-136-199818-s209.pdf]
